# Combining mechanistic and machine learning models for predictive engineering and optimization of tryptophan metabolism

**DOI:** 10.1038/s41467-020-17910-1

**Published:** 2020-09-25

**Authors:** Jie Zhang, Søren D. Petersen, Tijana Radivojevic, Andrés Ramirez, Andrés Pérez-Manríquez, Eduardo Abeliuk, Benjamín J. Sánchez, Zak Costello, Yu Chen, Michael J. Fero, Hector Garcia Martin, Jens Nielsen, Jay D. Keasling, Michael K. Jensen

**Affiliations:** 1 Novo Nordisk Foundation Center for Biosustainability, Technical University of Denmark, Kgs., Lyngby, Denmark; 2 Joint BioEnergy Institute, Emeryville, CA USA; 3 Biological Systems and Engineering Division, Lawrence Berkeley National Laboratory, Berkeley, CA USA; 4 DOE Agile BioFoundry, Emeryville, CA USA; 5 TeselaGen SpA, Santiago, Chile; 6 TeselaGen Biotechnology, San Francisco, CA USA; 7 Department of Biology and Biological Engineering, Chalmers University of Technology, Gothenburg, Sweden; 8 Novo Nordisk Foundation Center for Biosustainability, Chalmers University of Technology, Gothenburg, Sweden; 9 BCAM, Basque Center for Applied Mathematics, Bilbao, Spain; 10 BioInnovation Institute, Ole Maaløes Vej 3, DK-2200 Copenhagen N, Denmark; 11 Department of Chemical and Biomolecular Engineering & Department of Bioengineering, University of California, Berkeley, CA USA; 12 Center for Synthetic Biochemistry, Institute for Synthetic Biology, Shenzhen Institutes of Advanced Technologies, Shenzhen, China

**Keywords:** Metabolic engineering, Applied microbiology, Synthetic biology

## Abstract

Through advanced mechanistic modeling and the generation of large high-quality datasets, machine learning is becoming an integral part of understanding and engineering living systems. Here we show that mechanistic and machine learning models can be combined to enable accurate genotype-to-phenotype predictions. We use a genome-scale model to pinpoint engineering targets, efficient library construction of metabolic pathway designs, and high-throughput biosensor-enabled screening for training diverse machine learning algorithms. From a single data-generation cycle, this enables successful forward engineering of complex aromatic amino acid metabolism in yeast, with the best machine learning-guided design recommendations improving tryptophan titer and productivity by up to 74 and 43%, respectively, compared to the best designs used for algorithm training. Thus, this study highlights the power of combining mechanistic and machine learning models to effectively direct metabolic engineering efforts.

## Abstract (web summary)

In metabolic engineering, mechanistic models require prior metabolism knowledge of the chassis strain, whereas machine learning models need ample training data. Here, the authors combine the mechanistic and machine learning models to improve prediction performance of tryptophan metabolism in baker’s yeast.

## Introduction

Metabolic engineering is the directed improvement of cell properties through the modification of specific biochemical reactions^1^. Beyond offering an improved understanding of basic cellular metabolism, the field of metabolic engineering also envisions sustainable production of biomolecules for health, food, and manufacturing industries, by fermenting feedstocks into value-added biomolecules using engineered cells^2^. These promises leverage tools and technologies developed over recent decades that include both nonintuitive evolution-guided approaches, such as adaptive laboratory evolution^3,4^, as well as rational approaches combining mechanistic metabolic modeling, targeted genome engineering, and robust bioprocess optimization; ultimately aiming for accurate and scalable predictions of cellular phenotypes from deduced genotypes^5^.

Among the different types of mechanistic models for simulating metabolism, genome-scale models (GSMs) are one of the most popular approaches, as they are genome complete, covering thousands of metabolic reactions. These computational models not only provide qualitative mapping of cellular metabolism^6,7^, but have also been successfully applied for the discovery of metabolic functions^8^, and to guide engineering designs toward desired phenotypes^9^. As GSMs are built based only on the stoichiometry of metabolic reactions, several methods have been developed to account for additional layers of information, regarding the chemical intermediates and the catalyzing enzymes participating in the metabolic pathways of interest^10^. Nevertheless, all these mechanistic models require a priori knowledge, as well as high-quality data for accurate prediction^11,12^.

Machine learning (ML) provides a complementary approach to guide metabolic engineering by learning patterns on system behavior from large experimental datasets^13^. As such, ML models differ from mechanistic models by being purely data-driven. Indeed, ML methods for the generation of predictive models on living systems are becoming ubiquitous, including applications within genome annotation, de novo pathway discovery, product maximization in engineered microbial cells, pathway dynamics, and transcriptional drivers of disease states^14^. While being able to provide predictive power based on complex multivariate relationships^15^, the training of ML algorithms requires large datasets of high quality, and thereby imposes certain standards for the experimental workflows. For instance, for genotype-to-phenotype predictions, it is desirable that datasets contain a high variation between both genotypes and phenotypes^16^. Also, measurements on the individual experimental unit, e.g., a strain, should be accurate and obtainable in a high-throughput manner, in order to limit the number of iterative design–build–test–learn cycles needed to reach the desired output.

While mechanistic models require a priori knowledge of the living system of interest, and ML-guided predictions require ample multivariate experimental data for training, the combination of mechanistic and ML models holds promise for improved performance of predictive engineering of cells by uniting the advantages of the causal understanding of mechanism from mechanistic models, with the predictive power of ML^15,17^. Metabolic pathways are known to be regulated at multiple levels, including transcriptional, translational, and allosteric levels^13^. To cost-effectively move through the design and build steps of complex metabolic pathways, combinatorial optimization of metabolic pathways, in contrast to sequential genetic edits, has been demonstrated to effectively facilitate the searching for global optima for outputs of interest (i.e., production^18^). Searching global optima using combinatorial approaches involves facing an exponentially growing number of designs (known as the combinatorial explosion) and requires efficient building of multiparameterized combinatorial libraries. However, this challenge can be mitigated by using intelligently designed condensed libraries that allow uniform discretization of multidimensional spaces: e.g., by using well-characterized sets of DNA elements controlling the expression of candidate genes at defined levels as opposed to using more less-/non-characterized random elements^19,20^. As cellular metabolism is regulated at multiple levels^21,22^, an efficient search strategy for global optima using combinatorial approaches should also take this into consideration, e.g., by using mechanistic models, “omics data repositories”, and a priori biological understanding. Still it should be noted, that even with intelligent choice of design parameters and efficient library construction, there is no guarantee mathematical models will reach such a global optimum.

Here we combine mechanistic and ML models to enable robust genotype-to-phenotype predictions as a tool for metabolic engineering. The approach is exemplified for predictive engineering and optimization of the complexly regulated aromatic amino acid (AAA) pathway that produces tryptophan in baker’s yeast *Saccharomyces cerevisiae*^23^. We define a 7776-membered combinatorial library design space, based on five genes selected from GSM simulations and a priori biological understanding, each controlled by six different promoters from a set of 30 promoters selected from transcriptomics data mining. To train predictive models for tryptophan biosynthesis rate in yeast, we collect >124,000 experimental time series data points derived from fluorescent read-outs of an engineered tryptophan biosensor encoded into >500 different strain designs. This enable selection of optimal sampling time points, from that we explore fluorescence synthesis rates of ~3% (250/7,776) of the possible genetic designs of the library design space. Based on genotype data, growth profiles, and the biosensor output, we train various ML algorithms. Predictive models based on these algorithms identify designs exhibiting up to 74% higher tryptophan titers than best designs used for training the models.

## Results

### Model-guided design of high tryptophan production

One prime example of the multitiered complexity regulating metabolic fluxes is the shikimate pathway, driving the central metabolic route leading to AAA biosynthesis^24^. This pathway has enormous industrial relevance, since it has been used to produce bio-based replacements of a wealth of fossil fuel-derived aromatics, polymers, and potent human therapeutics^25^.

**Fig. 1:**
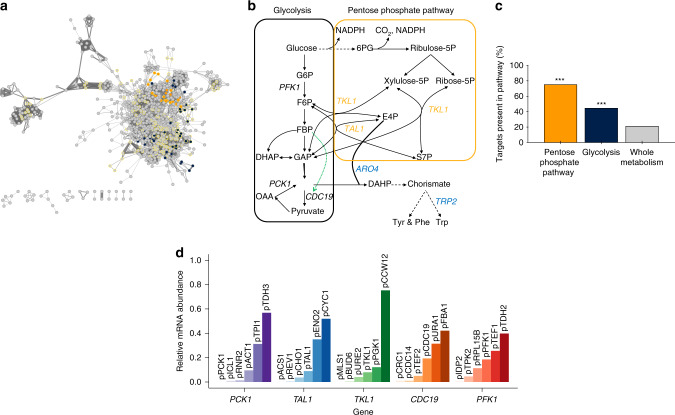
Gene targets and promoters for combinatorial engineering of tryptophan metabolism in *S. cerevisiae*. **a** Gene–gene interaction network built with Cytoscape, showing that pentose phosphate pathway and glycolysis are both in the core of metabolism in close proximity to many genes. Nodes are all 909 genes in yeast metabolism^67^, sharing connections based on the number of shared metabolites by the corresponding reactions that the genes are related to: the thicker the edge, the higher the number of shared metabolites. Currency metabolites such as water, protons, ATP, etc. are removed from the analysis. The prefuse force directed layout is used for displaying the network. Genes are highlighted with a yellow border if they are selected targets by the mechanistic modeling approach, and in orange and dark blue if they belong to the pentose phosphate pathway or glycolysis, respectively. **b** Simplified map of metabolism showing the selected gene targets from glycolysis (dark blue) and pentose phosphate pathway (orange) based on a combination of mechanistic genome-scale modeling and literature studies for optimizing tryptophan production. Black dashed lines indicate multistep reactions. Dashed green line indicates allosteric activation. G6P glucose 6-phosphate, F6P fructose 6-phosphate, FBP fructose 1,6-bisphosphate, GAP glyceraldehyde 3-phosphate, DHAP dihydroxyacetone phosphate, PEP phosphoenolpyruvate, OAA oxaloacetate, 6PG 6-phosphogluconate, E4P erythrose 4-phosphate, S7P sedoheptulose 7-phosphate, DAHP 3-deoxy-7-phosphoheptulonate, Tyr tyrosine, Phe phenylalanine, Trp tryptophan. **c** Percentage of genes in glycolysis (dark blue) and pentose phosphate pathway (orange) that were predicted by the mechanistic modeling to increase tryptophan production compared to the percentage of genes predicted as targets from the whole metabolism. ***P-value < 0.05, two-sided Fisher’s exact testing with *n* = 54 and 24 for the glycolysis and pentose phosphate pathway, respectively. **d** Relative messenger RNA (mRNA) abundance, calculated for each gene as the proportion of mRNA reads obtained for any given promoter relative to the total sum of mRNA reads from each bin of six promoters. Absolute abundances for the 30 promoters were measured in *S. cerevisiae* CEN.PK113-7D in the mid-log phase^32^. The promoters are grouped according to intended combinatorial gene associations. Source data underlying Fig. 1d are provided as a Source data file.

To search for gene targets to perturb tryptophan production, we initially performed constraint-based modeling for predicting single gene targets, with a simulated objective of combining growth and tryptophan production^26^. From this analysis, we retrieved 192 genes, covering 259 biochemical reactions, which showed considerable changes as production shifted from growth toward tryptophan production (Fig. 1a, b, Supplementary Data 1). By performing an analysis for statistical overrepresentation of genome-scale modeled metabolic pathways, we observed that both the pentose phosphate pathway (PPP) and glycolysis were among the top pathways with a significantly higher number of gene targets compared to the representation of all metabolic genes (Fig. 1c, Supplementary Data 2). Among the predicted gene targets in those pathways, *CDC19*, *TKL1*, *TAL1*, and *PCK1* were initially selected as targets for combinatorial library construction (Fig. 1b), as these genes have all been experimentally validated to be directly linked or to have an indirect impact on the shikimate pathway precursors erythrose 4-phosphate (E4P) and phosphoenolpyruvate (PEP). Specifically, *CDC19* encodes the major isoform of pyruvate kinase converting PEP into pyruvate to fuel the tricarboxylic acid cycle, while *TKL1* and *TAL1* that encode the major isoform of transketolase and transaldolase, respectively, in the reversible non-oxidative PPP, have been reported to impact the supply of E4P^27,28^. In addition, focusing on the E4P and PEP linkage, *PCK1* encoding PEP carboxykinase, was also selected due to its regeneration capacity of PEP from oxaloacetate^29^. Lastly, while not being predicted as a target by the constraint-based modeling approach, the *PFK1* gene, encoding the alpha subunit of heterooctameric phosphofructokinase (PFK1), catalyzing the irreversible conversion of fructose 6-phosphate to fructose 1,6-bisphosphate (FBP), was selected, as insufficient activity of this enzyme is known to cause divergence of carbon flux toward the PPP across different kingdoms^30,31^.

Next, we mined transcriptomics datasets for the selection of promoters to control the expression of the five target genes. Here, we focused on well-characterized and sequence-diverse promoters to ensure rational designs spanning large absolute levels of promoter activities, and limit the risk of recombination within strain designs and loss of any genetic elements, respectively^32,33^ (Supplementary Fig. 1). Together, this mining resulted in the selection of 25 sequence-diverse promoters, which together with the five promoters natively regulating the target genes constitutes the parts catalog for combinatorial library design (Fig. 1d, Supplementary Fig. 1, Supplementary Table 1).

### Creation of a platform strain for a combinatorial library

**Fig. 2:**
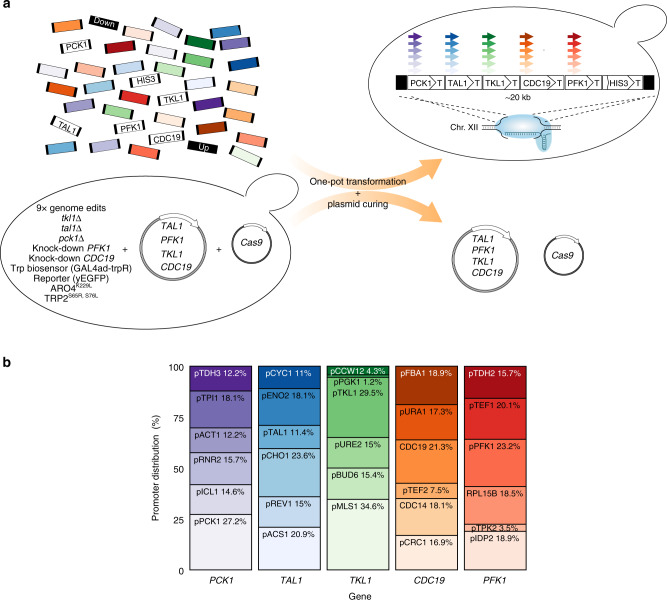
Construction and validation of the 13-parts assembled 20 kb combinatorial promoter:gene library. **a** Strategy for library construction including a 13-part in vivo assembly for the reintegration of target genes into a single genomic locus. The platform strain used for one-pot transformation includes a total of nine genome edits for knockout, knockdown, and heterologous expression of candidate genes (see “Methods” section). yEGFP yeast-enhanced green fluorescent protein. **b** Promoter distribution (name, % representation) by gene. Color intensity correlates with promoter strength (see Fig. 1d). Source data underlying Fig. 2b are provided as a Source data file.

To construct a combinatorial library targeting equal representation of 30 promoters expressing five target genes, we harnessed high-fidelity homologous recombination in yeast together with the targetability of CRISPR/Cas9 genome engineering for a one-pot assembly of a maximum of 7776 (6^5^) different combinatorial designs. Due to the dramatic decrease in transformation efficiency when simultaneously targeting multiple loci in the genome^34^, we targeted the sequential deletion of all five selected target genes from their original genomic loci, and next assembled a cluster of five expression cassettes into a single genomic landing as recently successfully reported for the single-locus glycolysis in yeast^35^ (Fig. 2a; see “Methods” section). However, as *CDC19* is an essential gene, and deletion of *PFK1* causes growth retardation^36,37^, our platform strain for library construction had a galactose-curable plasmid introduced expressing *PFK1*, *CDC19*, *TKL1*, and *TAL1* under their native promoters (see “Methods” section), after deleting *PCK1*, *TKL1*, and *TAL1*, and knocking down *CDC19* and *PFK1* (Fig. 2a). Prior to one-pot assembly of the combinatorial library, we integrated the two feedback-resistent shikimate pathway enzymes 3-deoxy-D-arabinose-heptulosonate-7-phosphate (DAHP) synthase (ARO4^K229L^) and anthranilate synthase (TRP2^S65R, S76L^) into the platform strain^38,39^, known to increase AAA accumulation in microbial cells^40,41^.

### One-pot construction of the combinatorial library

For library construction, we transformed in one-pot the platform strain with 38 different parts (30 promoters, 5 ORFs, *HIS3* ORF, and 2 homology regions) for 7776 unique 20 kb 13-parts assemblies at the targeted genomic locus (Fig. 2a). To assess assembly fidelity and ensure benchmarking, we also transformed yeast with five user-defined clusters, including one design with native promoters in front of each of the five selected genes (herein labeled the reference strain; Supplementary Table 2). Following transformation, we randomly sampled 480 colonies from the library, together with 27 colonies from the five control strains (507 in total), and successfully cured 423 out of 461 (92%) sufficiently growing strains of the complementation plasmid by means of galactose-induced expression of the dosage-sensitive gene *ACT1* (ref. ^42^; Fig. 2b, Supplementary Fig. 6). Next, genotyping identified 380 out of 461 (82%) of the sufficiently growing strains to be correctly assembled with only 9 out of 245 (3.7%) of the fully filtered library genotypes observed in duplicates (245 = 250 library and control genotypes—five control genotypes; Table 1, Supplementary Fig. 2). Based on a Monte Carlo simulation with 10,000 repeated samplings of 10,000 library colonies, and assuming percent correct assemblies and promoter distribution as determined for the library sample (Fig. 2), the expected number of unique genotypes among all library colonies was calculated to be 3759, equaling estimated library coverage of 48% (3759/7776). Importantly, all 30 promoters from the one-pot transformation were represented in the genotyped designs, with promoters *PGK1* (no. 14) and *MLS1* (no. 15), represented the least (1%) and most (35%), respectively (Fig. 2b).

**Table 1:** Key descriptive statistics for the library construction and genotyping.

Potential unique genotypes	7776
Number of library colonies	~10,000
Number of colonies sampled	480
Cured strains (%)	92
Correct assembly (%)	82
Repeated genotypes (%)	3.7

Taken together, these results demonstrate high transformation efficiency of the platform strain, high fidelity of parts assembly, and expected high coverage of the genetically diverse combinatorial library design.

### A biosensor for high-throughput library characterization

**Fig. 3:**
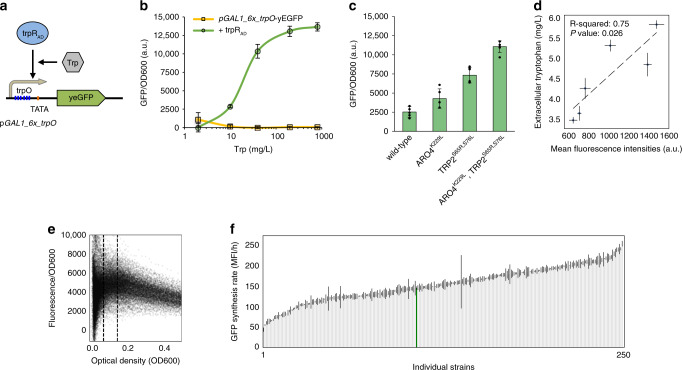
Phenotypic library characterization using an engineered tryptophan biosensor. **a** Schematic illustration of the design of the tryptophan (Trp) biosensor (trpR_AD_) engineered in this study. The trpR_AD_ indicates the engineering tryptophan biosensor composed of the *E. coli* TrpR fused to the GAL4 activation domain. The biosensor regulates an engineered reporter (yeGFP) *GAL1* promoter, including 6× copies of TrpR binding sites (*trpO*), placed upstream of the TATA box of *GAL1* promoter (*pGAL1_6x_trpO*). **b** Fluorescence normalized by optical density (OD_600_) for two strains related to concentration of tryptophan supplemented media (mean fluorescence intensity/OD, MFI/OD with standard errors, *n* = 3 biological replicates). Both strains contain the yeGFP reporter under the control of the *pGAL1_6x_trpO* reporter promoter, and only one strain expresses the Gal4 activation domain fused to trpR (in green). **c** Fluorescence normalized by OD_600_ for a wild-type strain and strains with expression of feedback-resistant versions of ARO4 and *TRP2*, *ARO4*^K229L^ and *TRP2*^S65R,S76L^, respectively (mean fluorescence intensity, MFI/h with standard errors, *n* = 4–5 biological replicates). **d** Extracellular tryptophan normalized by OD_600_ related to fluorescence normalized by OD_600_ (mean values with standard errors, *n* = 3 technical replicates).The *p*-value showing a significant slope is from a two-sided *t*-test performed on mean values for the six different genotypes. **e** Fluorescence divided by OD_600_ related to OD_600_ for library and control strains. Dashed lines are shown at OD_600_ equals 0.075 and 0.15. **f** Measured mean green fluorescent protein synthesis rate. MFI/h with standard errors, *n* = 3 technical replicates. The data is ranked according to increasing mean rate. The strain with five native promoters expressing the five candidate genes is highlighted in green. GFP green fluorescent protein, MFI mean fluorescence intensity, OD_600_ optical density (600 nm), a.u. arbitrary units. Source data underlying Fig. 3b–f are provided as a Source data file.

In order to support high-throughput analysis of tryptophan accumulation in library strains, we harnessed the power of modular engineering allosterically regulated transcription factors as small-molecule biosensors^43^. Here, a yeast tryptophan biosensor was developed based on the *trpR* repressor of the *trp* operon from *Escherichia* *coli*^44^. We first tested trpR-mediated transcriptional repression by expressing *trpR* together with a GFP reporter under the control of the strong *TEF1* promoter, containing a palindromic consensus *trpO* sequence^45^ (5′-GTACTAGTT-AACTAGTAC-3′) downstream of the TATA-like element^46^ (TATTTAAG; Fig. 3a). From this, we observed that *trpR* was able to repress GFP expression by 2.4-fold (Supplementary Fig. 3a). Next, to turn the native *trpR* repressor into an activator with positively correlated biosensor-tryptophan readout, we fused the Gal4 activation domain to the N-terminus of *trpR* (*GAL4*_*AD*_*-trpR*) under the control of the weak *REV1* promoter (Supplementary Fig. 3). For the reporter promoter, we placed *trpO* 97 bp upstream of the TATA-like element of the *TEF1* promoter (Supplementary Fig. 3b) and observed that *trpR* was able to activate GFP expression by a maximum of 1.75-fold upon supplementing tryptophan to the cultivation medium (Supplementary Fig. 3b). To further optimize the dynamic range of the reporter output, the GFP reporter was expressed under a hybrid promoter consisting of tandem repeats of triple *trpO* sequences (i.e., in total 6× *trpO* sequences) located 88 bp upstream of the TATA box in an engineered *GAL1* core promoter without Gal4 binding sites, ultimately enabling *GAL4*_*AD*_*-trpR*-mediated biosensing with a dynamic output range of fivefold, and an operational input range spanning supplemented tryptophan concentrations from ~2–200 mg/L (Fig. 3b).

To further validate the designed biosensor, we measured fluorescence output in strains engineered for expression of feedback-resistant versions of *ARO4* and *TRP2* (refs. ^38,39^; *ARO4*^K229L^ and *TRP2*^S65R,S76L^), and observed high biosensor outputs from these strains in line with previously demonstrated high enzyme activities in strains expressing *ARO4*^K229L^ and *TRP2*^S65R,S76L^ (refs. ^38,39^), and thus corroborating the ability of the tryptophan biosensor to monitor changes in endogenously produced tryptophan pools (Fig. 3c). Most importantly, we confirmed the biosensor readout as a valid proxy for tryptophan levels, by comparing external tryptophan titers measured by HPLC with a change in GFP intensities for six library strains spanning 2.5-fold changes in GFP intensities (*R*^*2*^ = 0.75; Fig. 3d).

Having established a biosensor for high-throughput screening of the combinatorial library, we next sought to explore the maximal resolution of the biosensor readout at the single-design level of growing isoclonal strains, with the intention to define optimal data sampling time point. To do so, we measured time series data of OD and GFP at 82 time points in triplicates for all 507 colonies (that is 480 from the library and 27 from the control strains), covering a total of 124,722 data points (Supplementary Figs. 4 and 5). Here, as we observed that the fluorescence per cell generally stabilized at an OD value of 0.075 and started to decrease beyond an OD value of 0.15 (Fig. 3e, Supplementary Fig. 4, see “Methods” section), and the between strains variation in fluorescence at the single-cell level was relatively high within this OD interval, we chose this interval for determining the GFP synthesis rate as a proxy for tryptophan biosynthesis rate. The average GFP synthesis rate of all quality-controlled strains (see below) was observed to vary between 43.7 and 255.7 MFI/h (approximately sixfold; Fig. 3f), with an average standard error of the mean of 6.6 MFI/h corresponding to an average coefficient of variation for the mean values of 4.3%. By comparison, the GFP synthesis rate of the platform strain, expressing *ARO4*^K229L^ and *TRP*2^S65R, S76L^ together with all five candidate genes under native promoters, was 144.8 MFI/h (Fig. 3f).

### Using machine learning to predict metabolic pathway designs

Having successfully established a combinatorial genetic library and a large phenotypic dataset thereof, we next assessed the potential of using ML to predict promoter combinations expected to improve tryptophan productivity. Since there is no single algorithm that is optimal for all conceivable general learning tasks^47^, we decided to improve our chances by using two different ML approaches for the single regression learning task of predicting promoter combinations controlling five genes that best improve GFP biosynthesis rates, as a proxy for tryptophan productivity: the Automated Recommendation Tool (ART) and EVOLVE algorithm^48,49^ (see “Methods” section). Briefly, ART uses a Bayesian ensemble approach where eight regressors from the scikit-learn library^50^ are allowed to vote on a prediction with a weight proportional to their accuracy; the EVOLVE algorithm is inspired by Bayesian Optimization and uses an ensemble of estimators as a surrogate model that predicts the outcome of the process to be optimized (see “Methods” section). As the quality of the data is of paramount importance for ML predictions, data were initially filtered in order to avoid strains (i) with insufficient growth, (ii) without sequencing data, (iii) with incorrect assembly, (iv) without plasmid curation, or (v) that exhibited more than one genotype (see “Methods” section; Supplementary Fig. 5). Following this, ~58% (266/461) of the growing strains remained after filtering, while another 3% of the remaining data were removed because of lack of reproducibility (high error in triplicate measurements), ultimately leaving high-quality sequencing and GFP data from 250 genotypes as input training dataset (Supplementary Fig. 5).

**Fig. 4:**
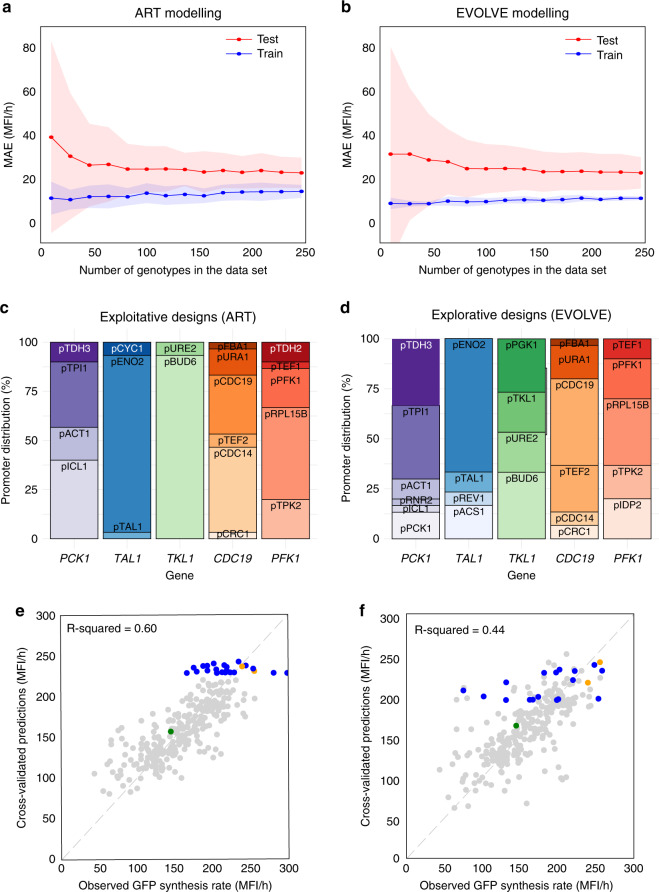
Machine learning-guided predictive engineering of tryptophan metabolism. **a**, **b** Learning curves for ART and EVOLVE algorithms, respectively. Mean absolute error (MAE) from model training and testing as a function of the number of genotypes in the dataset. Shaded areas represent 95% confidence intervals based on ten random samples of the given no of genotypes (*n*). Blue curves indicate MAE when calculated for the whole dataset (train), while red curves indicate the cross-validation, i.e., by training the models on 80% of the data and then testing the predictions of this model against measurements for the remaining 20% (test). **c**, **d** Promoter distributions for the 30 recommendations of the exploitative (ART) and explorative (EVOLVE) approach, respectively. The orders and colors of promoters correspond to those in Fig. 1c. **e**, **f** Cross-validated predictions vs average of measured GFP synthesis rate for the exploitative (ART) and explorative (EVOLVE) approach, respectively. Data are shown for library and control strains (gray markers; green markers show the platform strain expressing *ARO4*^K229L^ and *TRP2*^S65R,S76L^), as well as for recommended strains (blue markers; orange markers show recommendations that overlap between the two approaches). *R*-squared values are for cross-validated predictions for the whole dataset (not only training set data). MFI mean fluorescence intensity. Source data are provided as a Source data file.

Both modeling approaches, ART and EVOLVE, were able to recapitulate the data they were trained on. The average (obtained from ten independent runs) training mean absolute error (MAE) of the predicted tryptophan production compared to the measured values was 13.8 and 11.9 MFI/h for the ART and EVOLVE model approaches, respectively, when calculated for the whole dataset (Fig. 4a, b). These MAEs represent ~7 and 6% of the full range of measurements (50–200 MFl/h). The train MAE uncertainty (represented by the shaded area in Fig. 4a, b and quantified as the 95% confidence interval from ten runs) decreased slightly with increasing size of the training dataset for ART, whereas the overall uncertainty was smaller for the EVOLVE model approach (Fig. 4a, b). The ability to predict the production for new promoter combinations the algorithms had not been trained on was tested by cross-validation, i.e., by training the model on 90% of the data, and then testing the predictions of this model against measurements for the remaining 10% (tenfold cross-validation). Here, the average cross-validated MAE (test MAE) was 21.4 and 22.4 MFI/h for ART and EVOLVE model approaches, respectively (Fig. 4a, b), which represent ~11% of the full range of measurements. The test MAE decreased systematically with the size of the dataset, yet the decrease rate declined markedly as more data was added. However, while the two approaches had similar average cross-validated MAEs, the uncertainty of the MAEs was slightly smaller for ART than for the EVOLVE algorithm (Fig. 4a, b).

### Predictive engineering of high tryptophan production

Next, beyond enabling prediction of tryptophan production, we used an exploitative approach implemented in the ART model and an explorative one adopting the EVOLVE algorithm to recommend two sets of 30 prioritized designs aiming for high tryptophan production (Supplementary Tables 3 and 4). The exploitative model focuses on exploiting the predictive power to recommend promoter combinations that improve production, whereas the exploratory model combines predictive power with the estimated uncertainty of each prediction, to recommend promoter combinations^48,49^.

Among the recommendations from each of the two ML approaches, two overlapped (SP588 and SP627, Supplementary Tables 3 and 4). Interestingly, while use of *PGK1* promoter to control *TKL1* expression was underrepresented in the original library sample (Fig. 2b), the explorative set of recommendations included eight (even top three) designs with *PGK1* promoter for expression control of *TKL1*, and the exploitative approach included none (Supplementary Data 2; Fig. 4c, d). From construction of these recommendations, we used the same genome engineering approach as for library construction (Fig. 2a) to successfully construct 19 individual assemblies of the explorative recommendations and 24 individual assemblies of the exploitative recommendations. Interestingly, we were not able to construct any of the eight designs with the PGK1 promoter, partially explaining the lower number of viable strains found with the explorative approach.

Of the 41 recommendations constructed, the predictions from both sets generally fitted well with the measurements, and both approaches successfully enabled predictive strain engineering for high-performing GFP synthesis rates, with the best recommendation (SP606) having a measured GFP synthesis rate 106% higher than the already improved platform design (SP507), and 17% higher than the best one (SP271) in the library sample (Fig. 4e, f). This has been confirmed by HPLC analysis from small-scale deep-well batch cultivations of a diverse set of control, library, and recommended strains (Supplementary Fig. 7). We observed the strain SP606 having a 74 and 43% improvement in tryptophan titer and productivity, respectively, compared to the best strain design from the library sample (SP271). Moreover, eight recommendations were found in the top ten of productivity, of which four were from the exploitative set, three were from the explorative set, and one overlapping between the two sets. Comparing the output of the ART and EVOLVE approaches, the variation in measurements was higher for strains recommended with the explorative EVOLVE approach than for strains recommended with the exploitative ART approach (Fig. 4e, f), and the explorative approach included recommendations based on a more diverse set of promoters than the exploitative approach (Fig. 4c, d). Aligned with this, we observed that the recommendations from the EVOLVE approach also included a fraction of combinatorial designs with GFP synthesis rates below the reference strain (Fig. 4f). Still, taken together, when run in parallel, ART and EVOLVE approaches successfully enable predictive engineering of tryptophan biosynthesis strain designs, and for both approaches even strains with tryptophan biosynthesis rates beyond those previously observed for training the models (Fig. 4e, f, Supplementary Table 5 and 6).

## Discussion

In this study, we focus on the current possibility of using mechanistic and ML-guided models for predictive engineering of cellular metabolism as compared to sequential trial-and-error metabolic engineering iterations, or adaptive evolution-based reverse engineering for identification of nonintuitive changes. From this, we demonstrated that mechanistic and ML approaches can complement and enhance each other, enabling a more effective predictive engineering of living systems. Using a single design–build–test–learn cycle, this study (i) leveraged mechanistic GSMs to select and rank reactions/genes most likely to affect production, (ii) included the efficient one-pot construction of a library with different promoter combinations controlling the expression of these genes, and (iii) used ML algorithms trained on the ensuing phenotyping data to choose promoter combinations that further enhance tryptophan productivity. In total, we managed to increase tryptophan titers and productivity by up to 74% and 43%, respectively, compared to an already improved reference strain (*ARO4*^K229L^ and *TRP2*^S65R, S76L^).

To gather the large high-quality dataset required for ML approaches, we developed a biosensor that enabled the sampling of >124,000 GFP intensity measurements (82 time points) as a proxy for tryptophan flux for 1521 isoclonal designs (three replicates × 507 strains) in a high-throughput fashion, of which data from 250 strains were eventually used for successful training of ML algorithms (Fig. 3e, Supplementary Fig. 5a). Indeed, while requiring a few design iterations (Fig. 3a, Supplementary Fig. 3), the tryptophan biosensor ultimately allowed us to (i) phenotypically characterize an order of magnitude higher number of strains than in previous ML-guided metabolic engineering studies^20,51–53^, and (ii) identify optimal sampling points that displayed the largest differences between genotypes (Fig. 3c, Supplementary Fig. 4). Likewise, one-pot CRISPR/Cas9-mediated genome editing was a vital enabling technology for this project, since it allowed us to efficiently create a diverse 20-kb clustered combinatorial library with representation of all 30 specified sequence- and expression-diverse promoters to control five expression units, including very few duplicate designs (Fig. 2b, Table 1).

Enabled by this high-quality dataset, we used two different ML models for predicting productivity (ART and EVOLVE algorithm), and two different approaches to recommend strain designs (exploitative and explorative). Cross-validation showed that both models could be trained to show good correlations (MAE ~ 11% of the measurement range) between predictions and measurements for data they had not seen previously (test data). The test MAE decreased considerably with the number of genotypes in the dataset, this decrease was similar for both models. With this in mind, a relevant guideline for choosing a recommendation approach should focus on the desired outcome: the explorative approach providing a more diverse set of recommendations (Fig. 4c, d), whereas the exploitative approach provides less varied recommendations. We observed the largest improvement in titer and productivity when using the exploitative approach (Fig. 4e, f, Supplementary Fig. 7). However, if subsequent design–build–test–learn cycles are performed, the diversity of recommendations of the explorative approach could help avoid local optima of tryptophan production (Fig. 4e, f).

Notably, while the recommendations were able to improve biosynthesis rates, the predictions from both ML models were noticeably worse than for the library, reflecting the general challenge of extrapolating outside of the previous range of measurements. As such, we envision that future ML approaches will need to focus on models able to extrapolate more efficiently.

Another critical aspect to discuss from this study is the amount and quality of data needed, in order to increase the impact (e.g., improving titers, rates, and yields) and reduce model uncertainty. From this study, we argue that biosensors for time-resolved sensing of cellular metabolism not only enable sampling of large amounts of data points, but most importantly also facilitate the identification of a smaller sampling space for high-quality determination of metabolite biosynthesis rates (Fig. 3e). Specifically, we initially sampled triplicate measurements for 82 time points for all 576 strains, which when compared to growth, ultimately allowed us to select 15 time points of relevance for calculating maximal GFP biosynthesis rates. Likewise, while the one-pot library construction used in this study had an estimated coverage of 48% of the full combinatorial design space, the amount of strains used for training the algorithms only covered ~3%, yet enabled predictive engineering following a single design–build–test–learn cycle. This could be used to argue that more engineering iterations on even smaller datasets, potentially coupled to mixed exploitation and exploration approaches as recently demonstrated for cell-free production^54^, should be a valid avenue for ML-guided engineering of even less genetically tractable chassis, and for which no high-throughput screening method may even exist. With regards to this, we performed a follow-up test running the ART and EVOLVE approaches in explorative and exploitative modes, respectively. Here, we observed that the recommendations from EVOLVE in exploitative mode had overlaps of 20% (6/30) and 23% (7/30) to ART recommendations in exploitative, and EVOLVE recommendations in explorative mode, respectively. Complementary to this, the recommendations from ART in explorative mode only had overlaps of 3% (1/30) and 0% (0/30) to ART recommendations in exploitative and EVOLVE recommendations in explorative mode, respectively (Supplementary Fig. 8), indicating that the uncertainty of prediction of high GFP synthesis rate weighted differently for the two models in explorative mode.

While discovery of strain designs with titers and rates outcompeting previously reported high aromatics producers was not the main motivation for the study, it should be mentioned that all strains tested in this study produce much lower mg/L levels of tryptophan compared to previous studies, focusing on metabolic engineering and bioprocess optimization for aromatics overproduction (Fig. 3d, Supplementary Fig. 7)^23^. Indeed, as a suggestion for further optimization, it is possible that the reference strain used in this study is still subject to certain levels of feedback inhibition, as suggested by recent studies for AAAs derivatives^25,55^. Furthermore, the use of fed-batch cultivations as part of a bioprocess optimization would also be expected to enable cells to accumulate higher tryptophan titers compared to the titers obtained based on short batch cultivations in 96-well deep plates with low oxygen levels used in this study.

Despite the low production, there is still a positive correlation between tryptophan titer/productivity and the GFP synthesis rate (Fig. 3c, d, Supplementary Fig. 7), and the large-scale dataset from this study provides examples of results anticipated based on rational engineering, as well as nonintuitive predictions, enabling further advancement of the biological understanding of tryptophan metabolism. For instance, the best-performing strain (SP606, Supplementary Table 3 and Supplementary Data 3) predicted by ML, included knockdowns of both *CDC19* and *PFK1*, corroborating our intuitive strategies for increasing precursor availability: i.e., lower pyruvate kinase activity would lead to higher PEP pools, while limiting glycolysis redirects carbon flux into PPP and subsequently increases E4P^27^. Indeed, in bacteria, pyruvate kinase knockout has been used for the overproduction of shikimate pathway-derived aromatics products in bacteria^56–58^. Likewise, since yeast cells with *CDC19* deletion cannot grow on glucose^59^, dynamic silencing of *CDC19* and *PYK2* have been used for boosting production of para-hydroxybenzoic acid^60^, just as expression of a mutant *CDC19* pyruvate kinase with seemingly lower activity, in combination with overexpression of transketolase (*TKL1*), have been demonstrated to improve 2-phenylethanol production in yeast^61^. On the contrary, a similar strategy with lower *CDC19* activity, but in combination with *zwf1*∆ deletion (lacking the committed step toward the oxidative branch of PPP) was shown to reduce tyrosine titers^62^. Surprisingly, the top five strains predicted to have high tryptophan biosynthesis rates (SP606, SP616, SP624, SP588, and SPSP620, Supplementary Tables 3 and 4), all had low expression of *TKL1* and high expression of *TAL1*, despite the report that overexpression of *TKL1*, rather than *TAL1*, leads to higher AAA production in both *E. coli* and yeast^27,61^. These discrepancies remark the importance of carefully considering the systems-level context of these metabolic rules-of-thumb (e.g., overexpress *TKL1* instead of *TAL1* for higher amino acid production) to ensure their validity. Consistently, both the second (SP616) and third (SP624) best-performing strains, also predicted by ML, had low expression of *TKL1* and high expression of *TAL1*, together with very low expression (*TPK2* promoter) for *PFK1* and high expression of *CDC19*. One possible explanation is that, although normally expressed, the pyruvate kinase activity could be limited by the low level of its allosteric activator FBP due to the limited PFK expression. Another plausible explanation is that medium–high expression of *PCK1* (conversion of oxaloacetate to PEP) by *ACT1* or *TDH3* promoters in these two strains can replenish PEP pools consumed by pyruvate kinase. The fact that eight out of ten top-performing strains had high expression of *PCK1* (Supplementary Data 3), which was not predicted to be impactful on glucose by the GSM approach, indicates that this indeed has a positive effect on tryptophan biosynthesis rate, and stresses the importance of combining mechanistic and ML approaches.

Ultimately, in our case study, ML models have demonstrated good performance in predicting GFP biosynthesis rates for the training data designs (gray dots in Fig. 4e, f), while the recommended strains’ biosynthesis rates were less accurately predicted, likely because it involved an extrapolation effort that is a known weakness for ML methods (blue dots in Fig. 4e, f). In spite of this decrease in predictive power, the ML models can effectively recommend designs that improve tryptophan biosynthesis rates (Supplementary Fig. 7). However, this predictive power is heavily dependent on the availability of high-quality experimental data, which is not a prerequisite for mechanistic GSMs. Without any experimental input, GSMs are able to guide metabolic engineering using various constraint-based algorithms, which, however, predict a large number of potential targets and may also miss some effective ones (e.g., *PFK1* in our study), due to the lack of other information beyond metabolism, e.g., regulation in GSMs. To address this problem, manual efforts are currently needed to filter out less relevant targets and add intuitively promising ones based on existing knowledge. In addition, applying our approach to new models that enhance GSMs with more levels of information, such as kinetics^63^, gene expression^64^, and regulation^65^ is envisioned to further improve the model’s predictive power.

Irrespective of the ongoing efforts for model-guided engineering of living cells, this study highlights the enhanced predictive power from combining GSMs for selecting genetic targets with ML algorithms for leveraging experimental data. Finally, as even more efficient methods for combining data-driven ML algorithms and GSMs are developed, we envision accelerated improvements in our ability to engineer virtually any cell system effectively.

## Methods

### Experimental models

*S. cerevisiae* strains were derived from CEN.PK2-1C (EUROSCARF, Germany). These were cultivated in yeast synthetic dropout media (Sigma-Aldrich) at 30 °C. *E. coli* DH5α were cultivated in LB medium containing 100 mg/L ampicillin (Sigma-Aldrich) at 37 °C.

### Mechanistic modeling of high tryptophan flux

In order to select targets for increased tryptophan accumulation, we followed a constraint-based strategy implemented in a recent study^66^. Briefly, flux balance analysis (FBA)^26^ was used to simulate growth of *S. cerevisiae* at 11 different suboptimal growth conditions ranging from 30 to 80% of the maximum specific growth rate, with all remaining flux oriented toward tryptophan accumulation. Based on these simulations, a score was calculated for each reaction in metabolism as the average simulated flux fold change compared to maximum growth rate conditions. These reaction scores were in turn used to compute gene scores, by averaging the associated reaction scores. A gene score higher than one means that the gene is associated with reactions that increase in flux as tryptophan production increases and could point to a target for overexpression. On the other hand, a gene score lower than one signifies that the gene is connected to reactions that decrease their flux as tryptophan production increases, and therefore could be a target for downregulation. The analysis was performed with either glucose or ethanol as carbon sources, so to find candidates under a mixed-fermentation regime, a purely respiratory regime and the overlap between both regimes. The seventh version of the consensus GSM of *S. cerevisiae*^67^, a parsimonious FBA approach^68^, and the COBRA toolbox^69^ v. 3.0.6 were used for all simulations.

### Promoter selection

Each of the five gene targets was expressed under six unique promoters. The six promoters included the promoter native to the gene, as well as five promoters chosen to span a wide expression range All promoters were chosen based on absolute mRNA abundances measured for *S. cerevisiae* CEN.PK113-7D in the mid-log phase^32^, and unless otherwise stated were 1 kb in length by default. To minimize homologous recombination during one-pot transformation for library construction and potential loop out of promoters and genes following genomic integration, all scanned promoter sequences were aligned to ensure there were no extensive homologous sequence stretches.

### General strain construction

Strains were edited using the CasEMBLR method^70^. All integrations were directed toward EasyClone sites^71^. Homology regions between DNA parts were by default 30 bp, and homology regions, framing the repair assembly, were ~0.5 kb. Yeast transformations were performed by LiAc/SS carrier DNA/PEG method^70^. DNA parts and plasmids were purified using kits from Macherey-Nagel. PCR products for USER assembly were amplified using Phusion U Hot Start PCR Master Mix (ThermoFisher), bricks for transformation by Phusion High-Fidelity PCR Master Mix with HF Buffer (ThermoFisher), whereas colony PCRs were performed using 2× OneTaq Quick-Load Master Mix with Standard Buffer (New England Biolabs). Genomic DNA was extracted from overnight cultures using Yeast DNA Extraction Kit (Thermo Scientific). Oligos were purchased from IDT. Sequencing was performed by Eurofins. All primers, plasmids, and yeast strains, are listed in Supplementary Data 4 and Supplementary Tables 7 and 8, respectively.

### Platform strain construction

As *CDC19* is an essential gene, and deletion of *PFK1* causes growth retardation^36,37^, this genetic background was deemed unsuitable for efficient one-pot transformation. For this reason our platform strain for library construction had a galactose-curable plasmid introduced expressing *PFK1*, *CDC19*, *TKL1*, and *TAL1* under their native promoters, before performing two sequential rounds of CRISPR-mediated genome engineering to delete *PCK1*, *TKL1*, and *TAL1*, and knockdown *CDC19* and *PFK1* using the weak promoters *RNR2* and *REV1*, respectively (Fig. 2a). Moreover, several enzymes within the AAA biosynthesis are subject to allosteric regulations. Specifically, DAHP synthase (encoded by *ARO4*), which controls the entry of the shikimate pathway, is feedback inhibited by all three AAAs, although to different extents^72,73^. Anthranilate synthase (encoded by *TRP2*), which catalyzes the first committed step toward the tryptophan branch, is also inhibited by its end product tryptophan^24^. To maximize the transcriptional regulatory effect on the tryptophan flux, and benchmark with current state-of-the-art in shikimate pathway optimization, feedback-resistant variants of these two enzymes, *ARO*4^K229L38^ and *TRP2*^S65R, S76L39^, were overexpressed under the *TEF1* and *TDH3* promoters, respectively, at EasyClone site XI-3 (ref. ^71^) (Supplementary Table 8). Lastly, a tryptophan biosensor system was introduced by integrating corresponding sensor and reporter sequences into EasyClone sites at Chr. XI-2 and XI-5, respectively^71^.

### Construction of combinatorial library

Due to the dramatic decrease in transformation efficiency targeting multiple loci in the genome^34^, we opted for removing all five target genes from their original loci and assemble the five expression units into a single cluster for targeted integration into EasyClone site XII-5 (ref. ^71^), and thereby ensuring comparable genomic accessibility of all genes. While *PCK1*, *TKL1*, and *TAL1* were successfully knocked out, deleting *PFK1* and/or *CDC19* was unsuccessful. Alternatively, we replaced *PFK1* and *CDC19* promoters with weak *REV1* and *RNR2* promoters, respectively. Due to an expected loss of activity in PFK1 and pyruvate kinase (*CDC19*), and consequently slow ATP generation, the resulting strain (TrpNA-W) grew extremely poorly and was barely transformable using linear DNA fragments for assembly. To overcome this limitation, the TrpNA-W strain was complemented with plasmid pCfB9307 (Supplementary Table 8) harboring *PFK1*, *CDC19*, *TKL1*, and *TAL1* genes, which restored the growth to the wild-type level. The plasmid backbone carries yeast *ACT1* gene under the control of *GAL1* promoter, which can be used as counterselection of the plasmid due to the growth arrest caused by *ACT1* overexpression on galactose as the sole carbon source^42^ (Supplementary Fig. 6).

For combinatorial library construction, we adopted CasEMBLR^70^. Briefly, five target genes together with a *HIS3* expression cassette (in the order of *PCK1-TAL1-TKL1-CDC19-PFK1-HIS3*) were assembled in the same orientation and integrated at EasyClone site XII-5^71^. All five target genes (the complete ORFs) together with their terminators (500 bp downstream of the stop codon) were amplified from the genomic DNA of yeast strain CEN.PK113-7D using primers listed in Supplementary Data 4. All 30 promoters (defined as the 1000 bp upstream of the ORF) were amplified using primers with a 30 bp overlap to adjacent DNA parts (i.e., the terminator upstream and the target gene). All promoters can be found in Supplementary Table 1. The *HIS3* cassette was amplified from plasmid pRS413-HIS3 (ref. ^71^) with primers 30 bp overlapping with the *PFK1* terminator and fragment homologous to the downstream of XII-5. The *HIS3* cassette was included as one part of the assembly. The one-pot transformation of all 38 parts (30 promoters, 5 candidate genes, *HIS3* cassette, and up- and down-homology regions for EasyClone site XII-5) was performed with 50 mL the base strain grown to an optical density of 1.0 (equivalent to 6.5 mg of cell dry weight), 5.0 µg of plasmid expressing the guide RNA targeting XII-5, and 1.0 picomole of each of 13 DNA fragments. A total of 480 colonies were picked from ten transformation plates by dividing the area of each individual plate into four subareas of equal size and picking 12 colonies of varying size from each subarea.

Finally, the complementation plasmid introduced was cured by culturing strains to stationary phase twice in media with galactose instead of glucose as carbon source (Supplementary Fig. 6). The success of curing was then gauged by a growth assay where LEU auxotrophs were considered as cured and prototrophs as not cured. Control strains and recommended strains were constructed similarly to the library strains except that instead of transforming pools of promoter parts for each gene only specific promoters were transformed per gene.

### Development of tryptophan biosensor

The yeast tryptophan biosensor was developed based on the *trpR* repressor of the *trp* operon from *E. coli*^44^. The *trpR* gene was amplified from *E. coli* M1665 genome. All yeast promoters as well as the activator domain of *GAL4* were amplified from *S. cerevisiae* strain CEN.PK113-7D genome. All designs of trpR biosensor and GFP reporter were first cloned into the pRS416 (*URA3*) and pRS413 (*HIS3*) vectors, respectively, by USER cloning (NEB). The activator domain of *GAL4* (*GAL4*_AD_) was fused to trpR with a GSGSGS linker by USER cloning, and the expression of the gene product controlled by the weak *REV1* promoter. The *trpO* sequence was inserted into the *TEF1* promoter 8 bp downstream of the TATA-like element (TATTTAAG) by inverse PCR from a plasmid containing the P_*TEF1*_-*yEGFP-T*_*ADH1*_ cassette, with both primers containing the overhang AACTAGTAC (ie., half of the *trpO* sequence). The linear PCR product was treated with DpnI enzyme to fragmente the template plasmid and self-ligated to generate circular plasmid (Quick Ligation™ Kit, NEB). Promoters containing multiple *trpO* sequences were constructed by USER cloning from a synthetic DNA fragment (Integrated DNA Technologies) of a minimal *GAL1* promoter (−329 to −5 relative to the *GAL1* open reading frame, thus without the *GAL4* binding sequence that is located at −435 to −418) with 3× tandem repeats of *trpO* (separated by two nucleotides) inserted at 88 bp upstream of the TATA box (TATATAAA). Plasmids containing the sensor and reporter cassettes were transformed into yeast strain CEN.PK113-11C. To test the biosensor performance, yeast transformants were grown in selection media overnight and regrown in Delft medium supplemented with various tryptophan concentrations (2–1000 mg/L) for 6 h (typically reaching early exponential phase). GFP and mKate2 outputs were measured on Synergy MX microtiter plate reader (BioTek) with excitation/emission at 485/515 nm and 588/633 nm, respectively, and always normalized by absorbance at 600 nm (OD_600_). To construct the base strain for library assembly, the tryptophan sensor (P_*REV1*_-*GAL4*_*AD*_*-trpR-T*_*ADH1*_) and the reporter cassette (P_*GAL1core_3xtrpO*_-*yEGFP*-T_*ADH1*_, P_*TEF1_trpO*_-*mKate2*-T_*CYC1*_) were integrated into strain TC-3 (ref. ^34^) at the EasyClone sites XI-2 and XI-5^71^, respectively.

### Validation of biosensor by HPLC

To validate the correlation between biosensor reporter gene output and tryptophan production, we quantified extracellular tryptophan concentrations by HPLC^74^. Supernatants of cultivated strains were separated from the culture broth using AcroPrep Advance 96-Well Filter Plates (Pall Corporation) and centrifugation (5 min at 2200 × *g*) following 24 h of cultivation in synthetic dropout medium without tryptophan and histidine. From this, 200 µl was used for analysis on a Dionex 3000 HPLC system with a Zorbax Eclipse Plus C18 column (Agilent Technologies, Santa Clara, CA, USA). The column temperature was set to 30 °C. The flow rate was set to 1 ml/min with a mobile phase consisting of 0.05% acetate and a variable amount of acetonitrile. The total duration per sample was 12 min. This consisted of 10 min of separation, in which acetonitrile was reduced from 95 to 38.7% in 9.4 min and held for 0.6 min, 1 min of returning the acetonitrile concentration to 95%, and 1 min of holding the concentration till the end of the run. The injection volume was set to 10 µl. Elution of tryptophan was detected by UV at a wavelength of 280 nm. The data were processed using Chromeleon™ Chromatography Data System Software v7.1.3. Tryptophan concentrations were determined from a calibration curve. The specific tryptophan productivity was estimated as the average amount of tryptophan secreted into the medium per unit of biomass during the period of 1 day (μmol/gDCW/day).

### DNA sequencing of assembled clusters

Genomic DNA was extracted from overnight cultures using the LiOAc/SDS method adapted to a 96-well microtiter plate format. Each extract was used as a template in five PCR reactions spanning the five integrated promoters and amplifying from 1200 to 1700 bp. The PCR products were validated using a LabChip GX II (Perkin Elmer) and sequenced using PlateSeq PCR Kits (Eurofins) according to the manufacturer’s instructions. From the LabChip results, a PCR reaction was considered as trusted if it showed a strong band of the correct size; not trusted if it showed a strong band of the wrong size, and as no information (NI) gained if it showed a weak or no band. From the sequencing results, a sequencing reaction was considered as trusted if it showed an unambiguous sequence of the expected length (i.e., only limited by length of PCR fragment, stretches of the same nucleotide in the promoter or of ~1 kb limit of sanger sequencing reactions), not trusted if it showed an unambiguous sequence of the expected length with an assembly error, and NI gained if there were no or bad sequence results. If one or more sequencing results from the same strain showed double peaks in the promoter region the strain was considered as a double population. Finally, the promoter was noted as a failed assembly if either LabChip and or sequencing results were considered not trusted, as NI if the sequencing result was NI and else as the promoter predicted by pairwise alignment between sequencing results and promoter sequence.

### Measuring fluorescence and growth

Yeast cells were cultured O/N to saturation, diluted to OD_600_ 0.025 (measured by reading the absorbance at 600 nm on Synergy Mx Microplate Reader, BioTek) and then cultured again in a Synergy Mx Microplate Reader. While culturing, the reader measured OD_600_, and fluorescence with excitation and emission wavelengths of 485 and 515 nm, respectively, every 15 min for 20 h. All wells were sealed with VIEWseal membrane (Greiner Bio-One).

### Modeling and recommendation

All genotype and time series data as well as scripts for preprocessing are publicly available (seen “Data and Code availability” sections). Briefly, all OD_600_ and GFP measurements were subtracted background signals (i.e., mean value of OD_600_ and GFP measurements in wells containing pure media). Background signals were calculated for each 96-well plate. Strains were quality-controlled based on five criteria. The criteria were: (1) optical densities must cover the whole range up to 0.15 OD_600_ units to exclude uninoculated wells and wells with insufficient growth, (2) sequencing results must exist for all five promoter gene junctions, (3) the integrated sequence must be exactly as designed, (4) the complementation plasmid must be cured, and (5) the sequencing results must not indicate the presence of multiple genotypes (Supplementary Fig. 5a). Specific GFP synthesis rates were calculated as the difference in GFP divided by the difference in time (MFI/h) in the OD_600_ interval from 0.075 to 0.150, as measured by a Synergy Mx Microplate Reader from BioTek (a detailed description of the rationale behind this method can be found in connection with Supplementary Fig. 4).

In the ART approach, outliers were identified and removed based on replicate differences in GFP synthesis rate relative to the mean value for the strain. Replicates with the one percent most extreme differences were identified and the corresponding strains were removed. GFP synthesis rate was modeled as a function of promoter combination, represented through one-hot encoding, using the ART^48^. Briefly, ART uses a probabilistic ensemble model consisting of eight individual models. The weight of each ensemble model is considered a random variable with a probability distribution characterized by the available training data, and determined through Bayesian inference and Markov Chain Monte Carlo^75^. ART uses the trained ensemble model in combination with a Parallel Tempering approach^76^ to recommend 30 promoter combinations (unseen designs), which are predicted to improve production. The recommended designs were chosen as the 30 strains with the highest expected GFP synthesis rate predicted by the model. This recommendation approach was labeled exploitative since predictions with high uncertainty were not prioritized, although ART can provide both exploitative and explorative recommendations.

For the TeselaGen EVOLVE algorithm used in this study, outliers were identified and removed based on a method described by Rousseeuw and Hubert^77^. The decision was made on a per strain basis taking into account replicate to mean value differences. In cases where just a single replicate was left after filtering, this replicate was excluded as well. Of the remaining strains, GFP synthesis rates were modeled as a function of promoter combination coded as categorical variables using a TeselaGen-developed ML algorithm based on Bayesian Optimization^78^. The algorithm was set up to recommend 30 promoter combinations (unseen designs), and designs were chosen by highest selection score. The selection score was the expected improvement^79^, calculated based on predicted high GFP synthesis rate and the uncertainty of prediction. The approach was labeled explorative since high uncertainty weighed positively in the selection score calculation. While using EVOLVE for explorative recommendations, thereby complementing the ART approach, it should be mentioned that EVOLVE can be set up to provide both explorative and exploitative recommendations.

For both approaches, we tried encoding the promoter variables both as numbers ordered according to the counts from the RNAseq experiment (i.e., promoter strength^32^) and as one-hot encoding, and chose the one-hot encoding because it produced a lower MAE values and higher *R*-squared values.

### Reporting summary

Further information on research design is available in the Nature Research Reporting Summary linked to this article.

## Supplementary information

Supplementary Information

Peer Review File

Reporting Summary

Description of Additional Supplementary Files

Supplementary Data 1

Supplementary Data 2

Supplementary Data 3

Supplementary Data 4

## Data Availability

Data supporting the findings of this work are available within the paper and its Supplementary Information files. A reporting summary for this article is available as a Supplementary Information file. The datasets generated and analyzed during the current study are available from the corresponding author upon request. The genotype and time series datasets are available at The Joint BioEnergy Institute’s Inventory of Composable Elements (ICE; https://public-registry.jbei.org) and Experiment Data Depot (EDD; https://public-edd.jbei.org), respectively under the study “Zhang and Petersen, et al. 2019”. These are also available at GitHub (https://github.com/sorpet/Zhang_and_Petersen_et_al_2019). Source data are provided with this paper.

## Source data

Source Data

## Code availability

The FBA, with additional simulation details and filtering criteria, is available at GitHub (https://github.com/biosustain/trp-scores). The preprocessing and statistical calculations were made in a Python v. 3.6.5 environment with the packages seaborn 0.7.1, scikit-learn 0.20.2, pymc3 3.5, pandas 0.23.4, numpy 1.14.3, matplotlib 3.0.2, scipy 1.1.0, PTMCMC Sampler 2015.2, and ART. The process is documented in a jupyter notebook, available at GitHub (https://github.com/sorpet/Zhang_and_Petersen_et_al_2019). The notebook also contains the ART approach for model development and strain recommendation. The Teselagen software is available through commercial and non-commercial licenses (https://teselagen.com).
