# Supplementary material for: Combining mechanistic and machine learning models for predictive engineering and optimization of tryptophan metabolism
Source: Nat Commun. 2020 Sep 25;11:4880. doi: 10.1038/s41467-020-17910-1 (PMC7519671; doi:10.1038/s41467-020-17910-1)
Supplement: Supplementary file 1 — Supplementary Information [file 41467_2020_17910_MOESM1_ESM.pdf]

Combining mechanistic and machine learning models for predictive engineering and optimization of tryptophan metabolism

Zhang *et al.*

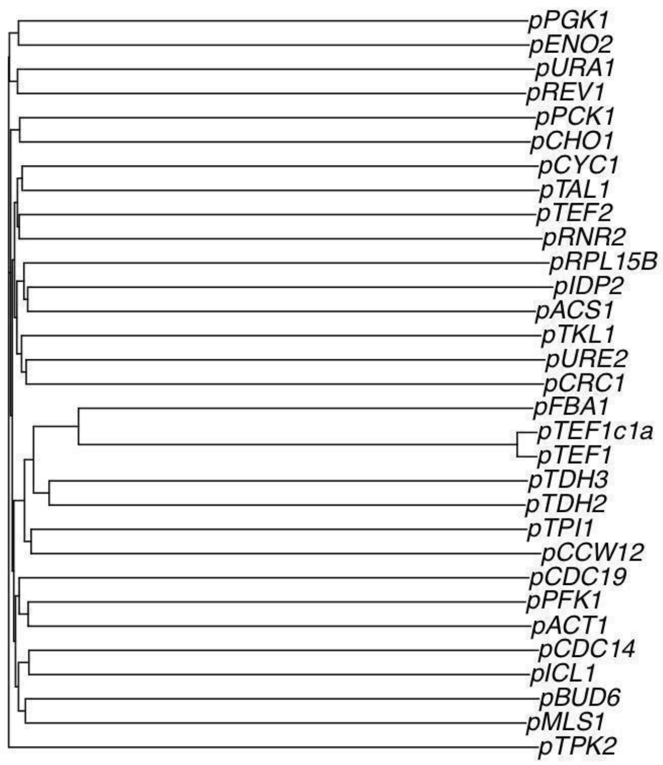

Supplementary Figure 1. Dendrogram of the sequence diversity of 30 selected native yeast promoters. Sequence pTEF1c1a with a single nucleotide change from pTEF1 has been added as a reference. The dendrogram was constructed using the neighbor-joining method[1,2].

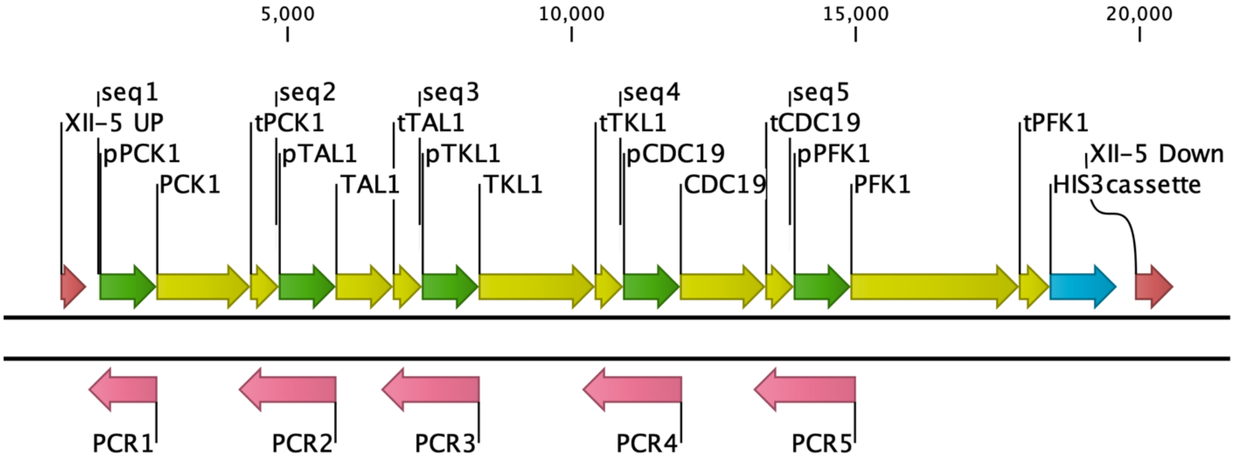

Supplementary Figure 2. Genotyping strategy. Schematic outline of the genotyping strategy to assess correct *in vivo* junction-junction assemblies of 11 parts, and the integration at EasyClone site XII-5[3]. Marked in red are chromosomal regions of EasyClone site XII-5, whereas green marks the promoters, and yellow the coding sequences and terminators. Marked in blue is the selectable *HIS3* expression cassette, while genotyping PCRs are marked in light red. Primers used for sequencing of the 5 PCR reactions are marked seq1-seq5. Related to Figure 1. Source data are provided as a Source Data file.

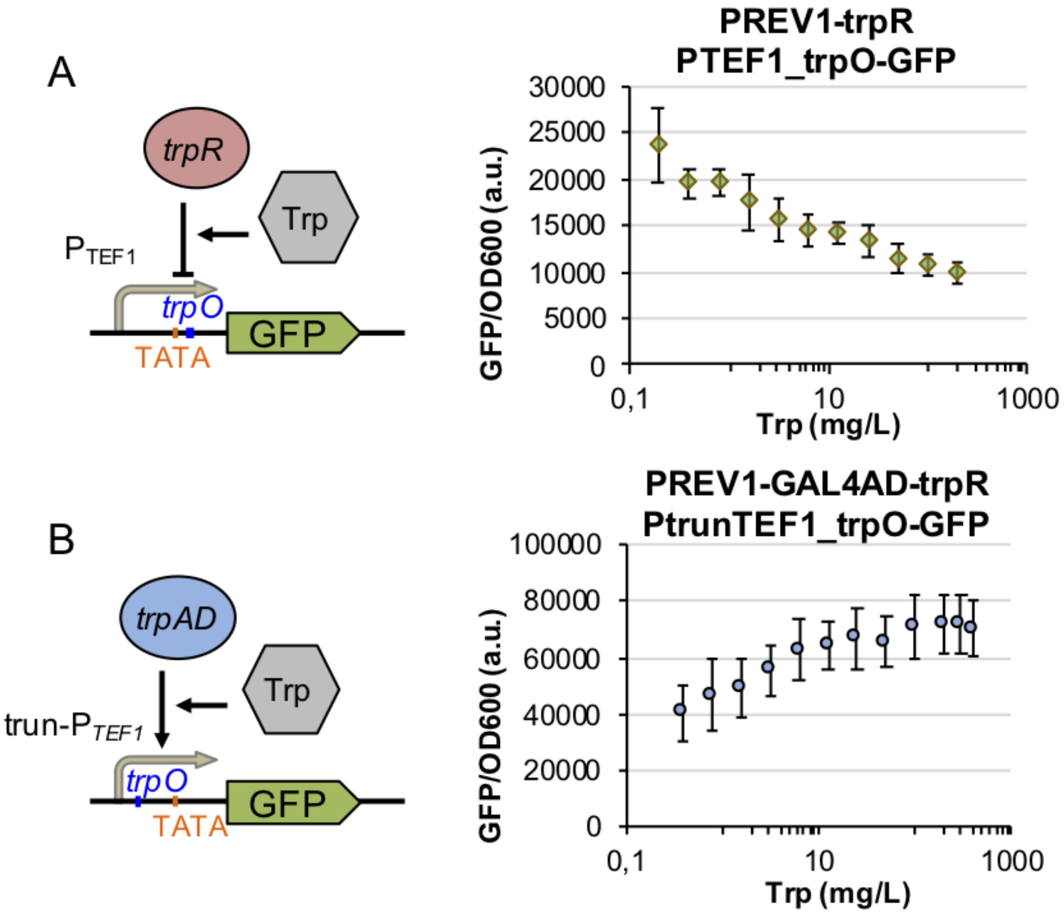

Supplementary Figure 3. Biosensor development and characterization. Overnight cultures of the strain containing sensor and reporter was used to inoculate fresh media supplemented with various concentrations of tryptophan and grown for 6 hours (early-mid exponential phase). Optical density (measured as absorbance at 600 nm)(OD600) was used to normalize green fluorescent protein (GFP) levels (excitation/emission at 485/515 nm). (**A**) *E. coli trpR* was directly expressed in a yeast strain harboring the yeast-enhanced GFP (yEGFP) reporter under the control of *TEF1* promoter containing *trpO* sequence inserted downstream of the TATA-like element. (**B**) The *trpR* gene was fused to the C-terminus of the activator domain of GAL4 ($GAL4_{ad}$) with a GSGSGS linker, turning this transcriptional repressor into an activator (trpAD). Accordingly, the *trpO* sequence was placed upstream of a truncated *TEF1* promoter (lacking region with multiple Rap1-binding sites). In (A-B) data are presented as mean values +/- SD for n = 3 biological replicates. Related to Figure 3. Source data are provided as a Source Data file.

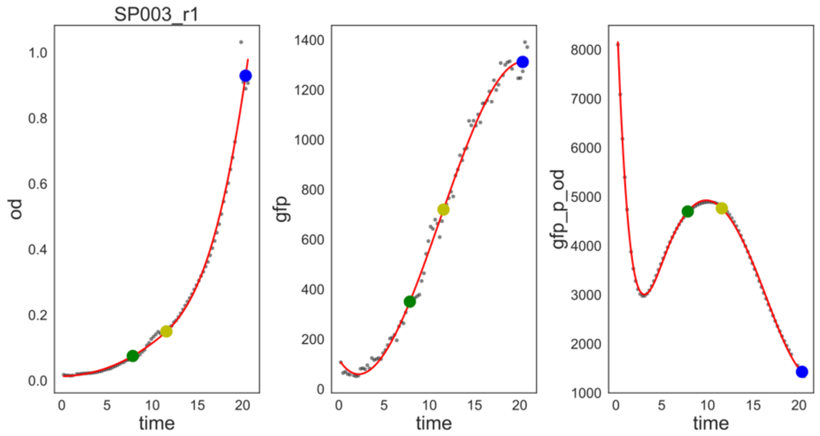

Supplementary Figure 4. Parameter estimation from time series data. Left panel - Representative growth curve of *S. cerevisiae* in microtiter plates (left panel). *S. cerevisiae* was grown in yeast synthetic drop-out media in 96-well microtiter plates, and cell density measured at optical density 600 nm (OD_{600}) over 20 hrs. Middle panel -Representative tryptophan biosensor output measured as fluorescence (GFP) in *S. cerevisiae* cells (n = 1). *S. cerevisiae* was grown in yeast synthetic drop-out media in 96-well microtiter plates, and green fluorescent protein (GFP) levels measured at 485 nm (OD_{485}) over 20 hrs. Right panel - Tryptophan biosensor output normalized by absorbance at 600 nm (OD_{600}) over 20 hrs. For all panels the red line shows model fitting using a univariate spline. All plots represent a single replicate measurement (n = 1). The green, yellow and blue markers indicate OD_{600} = 0.075, OD_{600} = 0.15, and maximum rate of OD_{600} increase, respectively. Related to Figure 3E-F.

When calculating GFP synthesis rates (increase in GFP/time) we normalized our measurements with the number of cells (GFP/OD_{600}/time), because it is not possible to inoculate the medium with exactly the same number of cells. In order to calculate normalized rates (GFP/OD_{600}) we measured both OD_{600} and GFP over time for all >500 strains. We only calculated rates in the period when GFP/OD_{600} was fairly constant and high (Figure 3E). Here, we observed that this was the case in the early part of the exponential phase, i.e. not in the entire exponential growth phase. From this, we observed that the increase in GFP/time declined before OD_{600}/time. This is considered to be due GFP maturation being more sensitive to oxygen than to cell growth. Picking the correct period for calculating rates was necessary to make sure that we got the actual strain characteristics, and not biases due to the specific laboratory setup (e.g. that the cells begin to shade one another at high OD_{600} and thereby limit detection of GFP, or due to feedback degradation of GFP). By ensuring this we achieved high reproducibility, and thus a higher signal to noise ratio (Figure 3F).

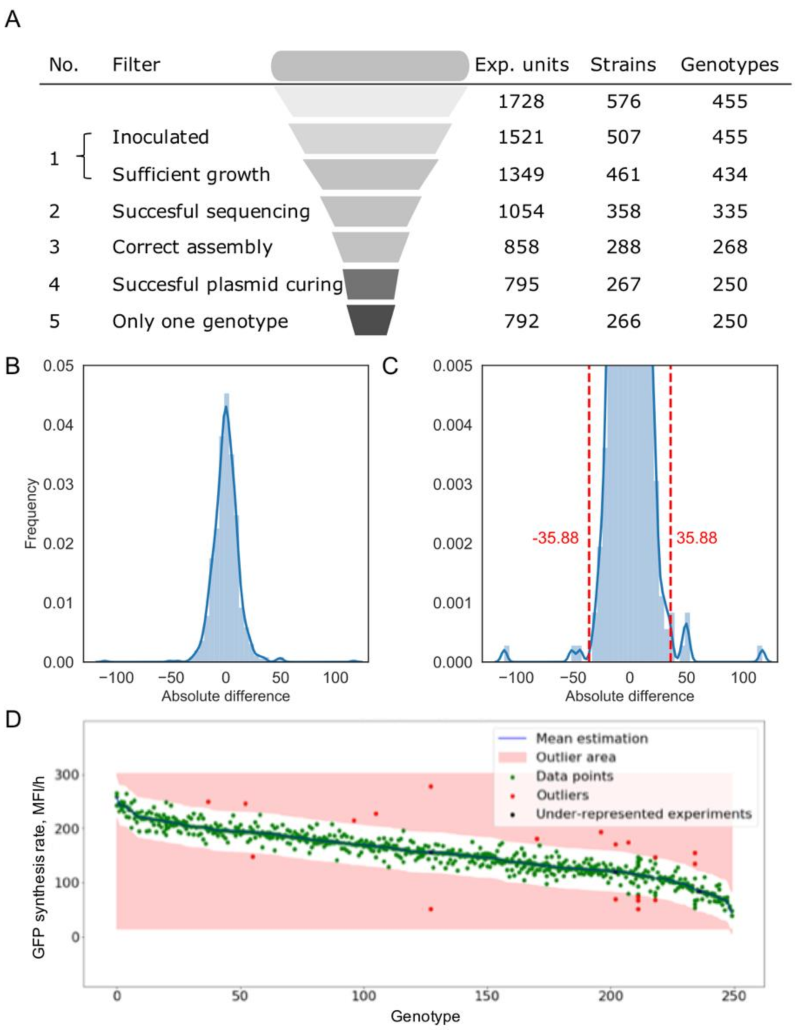

Supplementary Figure 5. Data filtering and outlier removal. (**A**) Schematic illustration of the various filtering steps applied for data quality control. The six steps used for filtering are indicated by number to the left and listed to the right are the numbers of unique genotypes as inferred from sequencing, the number of strains, and the number of experimental units (Exp. units, n = 3). (**B**) The distribution of absolute differences between replicate measurements (n = 3) of strain GFP synthesis rate. (**C**) Same as in (B), but with y-axis expanded by a factor 10. For (B-C) the dashed red lines delimit the 1% most extreme differences between replicates which were removed in the ART modelling approach. (**D**) GFP synthesis rate compared to strain genotype (n = 3). The data is ordered according to decreasing mean GFP synthesis rate. Data points included in the TeselaGen EVOLVE modeling approach are shown in green, whereas data points in red or black were excluded. Red markers indicate outliers whereas black markers indicate strains for which only one replicate is left after outlier removal. Related to Figures 3 and 4. Source data underying Supplementary Figure 5B and 5C are provided as a Source Data file.

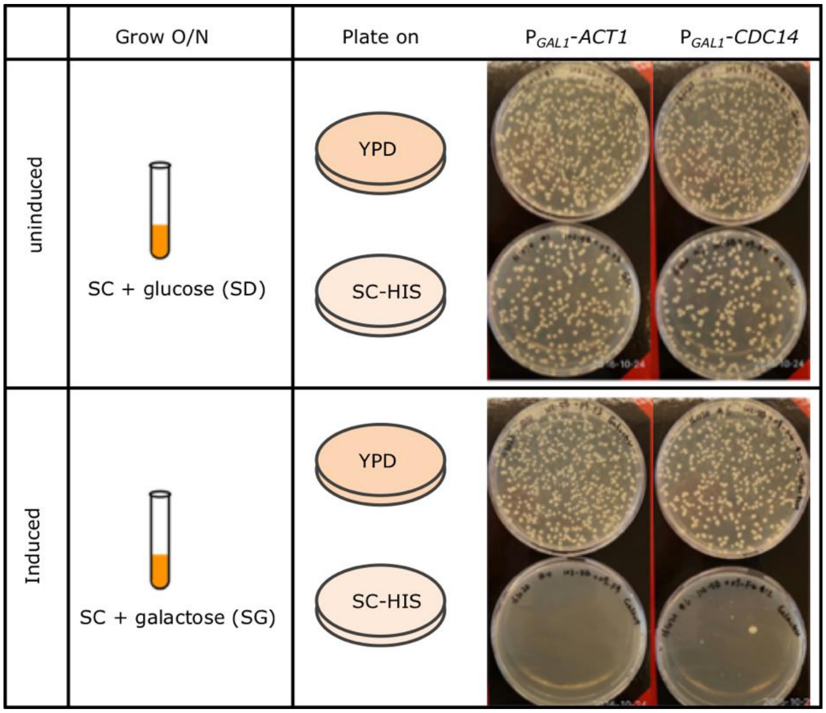

Supplementary Figure 6. Construction of an easy-curable plasmid using counter selection. Two dosage sensitive genes (*ACT1* & *CDC14*) were expressed under the control of the galactose-inducible *GAL1* promoter and cloned into USER vector pRS413-mKate2 (pCfB2866) [4]. To test the efficiency of counter selection, yeast strain with a plasmid containing one of the counter selection cassettes (pRS413-HIS3 P_{GAL1}-*ACT1*-T_{IDP1} or P_{GAL1}-*CDC14*-T_{ADH1}) was grown in both non-induction (synthetic complete + glucose) and induction (synthetic complete + galactose) media for 18 hrs. A diluted aliquot of culture was spread onto both YPD (without selection for the *HIS3* selectable marker) and SC-HIS (with selection for the *HIS3* selectable marker) drop out agar plates. Only cultures without growth on SC-HIS selective media were used for further studies. O/N = over-night.

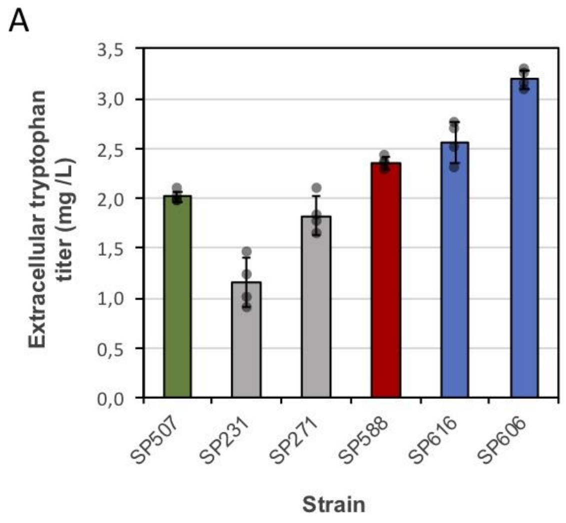

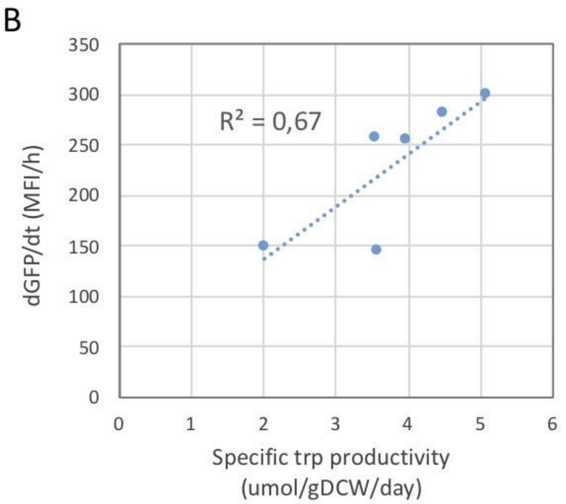

C

Strain	Extracellular trp titer (mg/L)	Final OD_{600}	Biomass conc. (gDCW/L)	Specific trp productivity (µmol/gDCW/day)	selection	dgfp/dt
SP507	2.02±0.05	21.2	2.75	3.59	library_mid	144.80
SP231	1.15±0.25	21.4	2.78	2.02	library_mid	147.82
SP271	1.83±0.19	19.3	2.51	3.57	library_high	255.65
SP588	2.35±0.06	22.2	2.89	3.99	tes_high	255.34
SP616	2.56±0.20	21.4	2.79	4.51	jbei_high	280.88
SP606	3.19±0.09	23.6	3.07	5.09	jbei_high	298.80

Supplementary Figure 7. Validation of tryptophan titers and productivities of selected strain designs. (**A**) Concentration of extracellular tryptophan measured by HPLC (mean +/- standard error; n=4 technical replicates) in wells of fixed size (i.e. titer) for 6 selected strains cultured for 24 hours in SD medium without histidine and tryptophan. The strains are: design with native promoters (SP507; green), library strains with middle or high GFP synthesis rate (SP231 and SP271; grey), EVOLVE explorative mode recommendation with high GFP synthesis rate (SP588; red) and ART exploitative mode recommendation with high GFP synthesis rates (SP616 and SP606; blue). Error bars indicate standard deviations of three biological replicates. (**B**) Correlation between measured mean specific GFP synthesis rate and the average specific tryptophan productivity (i.e., amount of tryptophan secreted per unit of biomass within 24 hours). (**C**) Values used for panels A-B. Biomass concentrations were converted from OD_{600} values using a correlation factor of 1 unit of OD_{600} = 0.13 gDCW/L (measured using the wild type yeast strain). dGFP = delta green fluorescent protein. dt = delta time. MFI = Mean fluorescence intensity. gDCW = gram dry cell weight. OD600 = Optical density (600 nm). Source data underlying Supplementary Figure 7A are provided as a Source Data file.

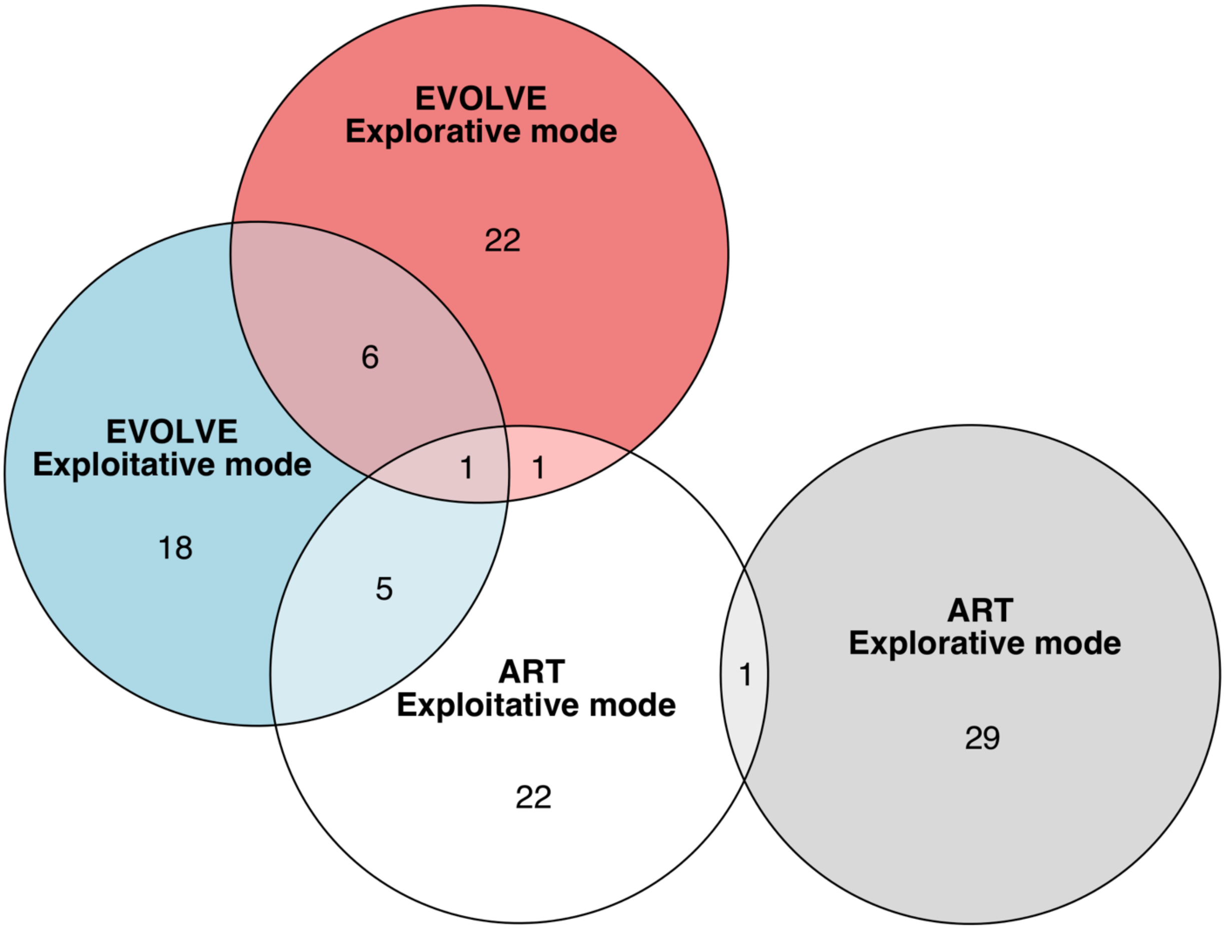

Supplementary Figure 8. Venn diagram of overlapping strain design recommendations. Overlap between the 30 recommendations given by the four different recommendation approaches: ART exploitative mode, ART explorative mode, EVOLVE exploitative mode, and EVOLVE explorative mode. Numbers shown represent numbers of overlapping designs. The designs recommended by the four approaches are listed in Supplementary Tables 3-6.

Supplementary Table 1. The 30 selected native yeast promoters, and their position in the combinatorial cluster.

Number	Name	Systematic name	Position in cluster
01	pPCK1	YKR097W	01
02	pTPI1	YDR050C	01
03	pICL1	YER065C	01
04	pRNR2	YJL026W	01
05	pACT1	YFL039C	01
06	pTDH3	YGR192C	01
07	pTAL1	YLR354C	02
08	pENO2	YHR174W	02
09	pACS1	YAL054C	02
10	pREV1	YOR346W	02
11	pCHO1	YER026C	02
12	pCYC1	YJR048W	02
13	pTKL1	YPR074C	03
14	pPGK1	YCR012W	03
15	pMLS1	YNL117W	03
16	pBUD6	YLR319C	03
17	pURE2	YNL229C	03
18	pCCW12	YLR110C	03
19	pCDC19	YAL038W	04
20	pURA1	YKL216W	04
21	pCRC1	YOR100C	04
22	pCDC14	YFR028C	04
23	pTEF2	YBR118W	04
24	pFBA1	YKL060C	04
25	pPFK1	YGR240C	05
26	pTDH2	YJR009C	05
27	pIDP2	YLR174W	05
28	pTPK2	YPL203W	05
29	pRPL15B	YMR121C	05
30	pTEF1	YPR080W	05

Note: Related to Figure 1.

Supplementary Table 2. Promoter combinations of library control strains.

Design	Position 1	Position 2	Position 3	Position 4	Position 5
1	1	7	13	19	25
2	6	12	18	23	28
3	4	11	17	24	30
4	2	8	14	23	28
5	3	9	15	20	26

Note: The numbers in each row refer to promoter numbers as shown in Supplementary Table 3. Design no. 1 contains the promoters that are native to the genes at the five positions. Related to Figure 1 and 3.

Supplementary Table 3. Top-30 promoter combinations as recommended by ART in exploitative mode.

Priority	Promoter	*PCK1*	Promoter	*TAL1*	Promoter	*TKL1*	Promoter	*CDC19*	Promoter	*PFK1*	Build	Strain	dgfp_dt	Predicted
1	pICL1		pENO2		pBUD6		pCDC14		pTPK2		Yes	SP609	234.72	242.4
2	pICL1		pENO2		pBUD6		pCDC19		pTPK2		No			240.3
3	pICL1		pENO2		pBUD6		pCDC14		pRPL15B		Yes	SP610	201.91	240.2
4	pICL1		pENO2		pBUD6		pCDC19		pRPL15B		Yes	SP605	215.80	238.1
5	pICL1		pENO2		pBUD6		pCDC14		pPFK1		Yes	SP607	244.19	237.5
6	pICL1		pENO2		pBUD6		pCDC19		pTDH2		Yes	SP603	193.35	237.5
7	pICL1		pENO2		pBUD6		pCDC14		pTDH2		Yes	SP608	187.92	237.2
8	pTDH3		pENO2		pBUD6		pCDC14		pRPL15B		Yes	SP627	239.33	236.4
9	pICL1		pENO2		pBUD6		pCDC19		pPFK1		Yes	SP602	219.01	235.9
10	pTPI1		pENO2		pBUD6		pCDC19		pPFK1		Yes	SP586	175.43	235.2
11	pTPI1		pENO2		pBUD6		pCDC14		pTPK2		No			234.4
12	pACT1		pENO2		pBUD6		pCDC14		pRPL15B		Yes	SP620	253.94	233.7
13	pTPI1		pENO2		pBUD6		pCDC19		pTPK2		No			233.3
14	pTPI1		pENO2		pBUD6		pCDC14		pPFK1		Yes	SP591	205.20	232.9
15	pTPI1		pENO2		pBUD6		pTEF2		pRPL15B		No			232.7
16	pTPI1		pENO2		pBUD6		pCDC14		pRPL15B		Yes	SP593	193.57	231.5
17	pICL1		pENO2		pURE2		pCDC14		pRPL15B		Yes	SP612	218.93	231.4
18	pTPI1		pENO2		pBUD6		pCDC19		pRPL15B		Yes	SP588	255.34	230.7
19	pTPI1		pENO2		pBUD6		pURA1		pPFK1		Yes	SP589	179.41	230.3
20	pACT1		pENO2		pBUD6		pCDC19		pRPL15B		Yes	SP617	218.75	230
21	pACT1		pENO2		pBUD6		pCDC14		pPFK1		Yes	SP618	212.79	229.4
22	pTPI1		pENO2		pBUD6		pTEF2		pTEF1		No			229.4
23	pICL1		pENO2		pBUD6		pFBA1		pTDH2		Yes	SP611	223.48	229.2
24	pACT1		pENO2		pBUD6		pCDC14		pTPK2		Yes	SP619	228.85	229.1
25	pTDH3		pCYC1		pBUD6		pURA1		pRPL15B		Yes	SP633	217.05	229.1
26	pTDH3		pCYC1		pURE2		pURA1		pRPL15B		No			228.9
27	pICL1		pTAL1		pBUD6		pCDC14		pRPL15B		Yes	SP601	166.32	228.7
28	pTPI1		pENO2		pBUD6		pURA1		pRPL15B		Yes	SP590	205.96	228.5
29	pACT1		pENO2		pBUD6		pCDC19		pTPK2		Yes	SP616	280.88	228.4
30	pICL1		pENO2		pBUD6		pCRC1		pRPL15B		Yes	SP606	298.80	228.2

Note: Size of color bars indicate promoter expression strength (see Figure 1), and column “dgfp/dt” shows measured mean specific rate of GFP synthesis. Related to Figure 1 and 4.

Supplementary Table 4. Top-30 promoter combinations as recommended by TeselaGen EVOLVE in explorative mode.

Priority	Promoter *PCK1*	Promoter *TAL1*	Promoter *TKL1*	Promoter *CDC19*	Promoter *PFK1*	Build	Strain	dgfp_dt	Predicted
1	pTDH3	pENO2	pPGK1	pCDC19	pRPL15B	No			229.9
2	pACT1	pENO2	pPGK1	pTEF2	pIDP2	No			214.9
3	pPCK1	pENO2	pPGK1	pCDC19	pRPL15B	No			223.5
4	pTDH3	pENO2	pURE2	pCDC19	pIDP2	Yes	SP628	219.7	219.4
5	pTDH3	pENO2	pBUD6	pCDC14	pRPL15B	Yes	SP627	239.3	216.2
6	pTPI1	pENO2	pPGK1	pURA1	pPFK1	No			216.4
7	pPCK1	pENO2	pPGK1	pTEF2	pTPK2	No			224.3
8	pTDH3	pACS1	pTKL1	pCDC19	pTPK2	Yes	SP630	162.6	195.2
9	pRNR2	pENO2	pBUD6	pCDC19	pRPL15B	Yes	SP614	202.0	232.2
10	pTDH3	pENO2	pBUD6	pCDC19	pTPK2	Yes	SP624	258.2	230.9
11	pTPI1	pENO2	pPGK1	pCDC14	pTPK2	No			202
12	pTDH3	pENO2	pBUD6	pCDC19	pTEF1	Yes	SP625	222.0	230.5
13	pICL1	pACS1	pTKL1	pTEF2	pPFK1	Yes	SP613	198.5	194.9
14	pTPI1	pENO2	pURE2	pCDC19	pRPL15B	No			234
15	pTPI1	pACS1	pTKL1	pCDC19	pPFK1	Yes	SP600	131.3	194.9
16	pTPI1	pTAL1	pBUD6	pCRC1	pIDP2	Yes	SP582	101.8	199.3
17	pTPI1	pENO2	pURE2	pTEF2	pPFK1	No			223.2
18	pTPI1	pENO2	pURE2	pCDC19	pIDP2	Yes	SP597	131.6	216.6
19	pTDH3	pENO2	pURE2	pCDC19	pRPL15B	Yes	SP629	247.9	238
20	pTDH3	pACS1	pPGK1	pFBA1	pRPL15B	No			197.1
21	pTPI1	pENO2	pPGK1	pTEF2	pIDP2	No			217.5
22	pTPI1	pENO2	pBUD6	pCDC19	pRPL15B	Yes	SP588	255.3	241.3
23	pACT1	pREV1	pTKL1	pCRC1	pTEF1	Yes	SP621	166.7	195.3
24	pPCK1	pENO2	pURE2	pCDC19	pPFK1	Yes	SP580	181.6	228.3
25	pTPI1	pTAL1	pBUD6	pURA1	pIDP2	Yes	SP581	173.9	198.8
26	pTDH3	pENO2	pBUD6	pURA1	pRPL15B	Yes	SP626	197.4	228.7
27	pTPI1	pENO2	pBUD6	pTEF2	pTPK2	No			219.1
28	pACT1	pREV1	pTKL1	pTEF2	pPFK1	Yes	SP622	253.3	196.5
29	pPCK1	pTAL1	pBUD6	pURA1	pRPL15B	Yes	SP577	75.0	206.6
30	pTDH3	pACS1	pTKL1	pURA1	pTEF1	Yes	SP631	200.4	195.9

Note: Size of color bars indicate promoter expression strength (see Figure 1), and column “dgfp/dt” shows measured mean specific rate of GFP synthesis. Related to Figure 1 and 4.

Supplementary Table 5. Top-30 promoter combinations as recommended by JBEI ART in explorative mode.

Priority	Promoter	*PCK1*	Promoter	*TAL1*	Promoter	*TKL1*	Promoter	*CDC19*	Promoter	*PFK1*	Predicted
1	pTPI1		pCHO1		pPGK1		pURA1		pRPL15B		189.3
2	pICL1		pACS1		pBUD6		pCDC19		pTPK2		202.9
3	pTPI1		pCHO1		pPGK1		pTEF2		pRPL15B		188.8
4	pTPI1		pCYC1		pPGK1		pURA1		pTEF1		178.1
5	pTDH3		pCYC1		pMLS1		pURA1		pRPL15B		155.8
6	pICL1		pACS1		pBUD6		pCDC14		pTPK2		206.0
7	pTPI1		pCYC1		pPGK1		pURA1		pRPL15B		190.3
8	pPCK1		pCYC1		pPGK1		pURA1		pRPL15B		184.1
9	pPCK1		pCHO1		pMLS1		pTEF2		pTPK2		113.4
10	pTPI1		pCHO1		pPGK1		pURA1		pTEF1		178.3
11	pTPI1		pENO2		pPGK1		pURA1		pRPL15B		202.7
12	pTPI1		pREV1		pPGK1		pTEF2		pRPL15B		178.3
13	pTPI1		pCYC1		pPGK1		pTEF2		pRPL15B		183.2
14	pICL1		pENO2		pTKL1		pCDC19		pTPK2		175.5
15	pTPI1		pCHO1		pPGK1		pCDC19		pRPL15B		182.9
16	pTPI1		pCHO1		pPGK1		pCDC14		pRPL15B		181.0
17	pACT1		pCYC1		pPGK1		pURA1		pTEF1		185.5
18	pPCK1		pCYC1		pURE2		pTEF2		pTPK2		169.3
19	pICL1		pACS1		pMLS1		pCDC19		pTPK2		126.0
20	pTPI1		pCHO1		pPGK1		pTEF2		pTEF1		180.2
21	pTPI1		pCHO1		pPGK1		pURA1		pIDP2		146.0
22	pPCK1		pCHO1		pPGK1		pTEF2		pRPL15B		172.9
23	pTPI1		pCHO1		pPGK1		pTEF2		pIDP2		146.0
24	pTPI1		pENO2		pPGK1		pTEF2		pRPL15B		207.5
25	pTPI1		pACS1		pPGK1		pTEF2		pRPL15B		186.0
26	pPCK1		pCHO1		pPGK1		pURA1		pRPL15B		172.6
27	pTDH3		pCYC1		pURE2		pURA1		pRPL15B		228.8
28	pACT1		pCYC1		pPGK1		pURA1		pRPL15B		193.6
29	pICL1		pACS1		pMLS1		pCDC14		pTPK2		125.7
30	pTPI1		pCYC1		pPGK1		pTEF2		pTEF1		172.7

Note: Size of color bars indicate promoter expression strength (see Figure 1), and column “Predicted” shows predicted mean specific rate of GFP synthesis.

Supplementary Table 6. Top-30 promoter combinations as recommended by TeselaGen EVOLVE in exploitative mode.

Priority	Promoter	*PCK1*	Promoter	*TAL1*	Promoter	*TKL1*	Promoter	*CDC19*	Promoter	*PFK1*	Predicted
1	pICL1		pENO2		pBUD6		pCDC19		pRPL15B		243.9
2	pPCK1		pENO2		pBUD6		pCDC19		pRPL15B		243.6
3	pTPI1		pENO2		pBUD6		pCDC19		pRPL15B		243.3
4	pACT1		pENO2		pBUD6		pCDC19		pRPL15B		240.4
5	pTDH3		pENO2		pURE2		pCDC19		pRPL15B		238.0
6	pTDH3		pENO2		pBUD6		pTEF2		pRPL15B		237.5
7	pTDH3		pENO2		pBUD6		pCDC19		pPFK1		237.2
8	pTPI1		pENO2		pURE2		pCDC19		pRPL15B		234.0
9	pTDH3		pENO2		pBUD6		pCRC1		pRPL15B		234.0
10	pTDH3		pENO2		pURE2		pCDC19		pPFK1		233.7
11	pICL1		pENO2		pBUD6		pTEF2		pRPL15B		232.5
12	pACT1		pENO2		pBUD6		pTEF2		pRPL15B		232.4
13	pTDH3		pENO2		pBUD6		pCDC19		pIDP2		232.3
14	pRNR2		pENO2		pBUD6		pCDC19		pRPL15B		232.2
15	pPCK1		pENO2		pBUD6		pTEF2		pRPL15B		232.2
16	pICL1		pENO2		pURE2		pCDC19		pRPL15B		232.1
17	pTDH3		pENO2		pBUD6		pTEF2		pPFK1		231.7
18	pTPI1		pENO2		pBUD6		pTEF2		pRPL15B		231.7
19	pTDH3		pENO2		pBUD6		pCDC19		pTDH2		231.7
20	pTPI1		pENO2		pBUD6		pCDC19		pPFK1		231.3
21	pPCK1		pENO2		pURE2		pCDC19		pRPL15B		231.2
22	pACT1		pENO2		pURE2		pCDC19		pRPL15B		230.9
23	pTDH3		pENO2		pBUD6		pCDC19		pTPK2		230.9
24	pPCK1		pENO2		pBUD6		pCDC19		pPFK1		230.7
25	pICL1		pENO2		pBUD6		pCDC19		pPFK1		230.7
26	pTDH3		pENO2		pBUD6		pCDC19		pTEF1		230.5
27	pPCK1		pENO2		pBUD6		pCRC1		pRPL15B		230.4
28	pTDH3		pENO2		pURE2		pCDC19		pTPK2		230.0
29	pTDH3		pENO2		pPGK1		pCDC19		pRPL15B		229.9
30	pACT1		pENO2		pBUD6		pCRC1		pRPL15B		229.3

Note: Size of color bars indicate promoter expression strength (see Figure 1), and column “Predicted” shows predicted mean specific rate of GFP synthesis.

Supplementary Table 7. Plasmids constructed and used in study.

Name	Description	Reference
**Tryptophan biosensor development**		
pCfB4107	CEN6/ARS4 pRS413U-*HIS3*, P_{TEF2_trpO}-*yEGFP*-T_{ADH1}	This study
pCfB4108	CEN6/ARS4 pRS416U-*HIS3*, P_{REV1}-*trpR*-T_{ADH1}	This study
pCfB4743	CEN6/ARS4 pRS416U-*URA3*, P_{REV1}-*GAL4*$_{ad}$_*trpR*-T_{ADH1}	This study
pCfB4747	CEN6/ARS4 pRS416U-*URA3*, P_{REV1}-*GAL4*$_{ad}$-T_{ADH1}	This study
pCfB4750	CEN6/ARS4 pRS413U-*HIS3*, P_{TEF2}-*mKate2*-T_{IDP1}, $P_{trunTEF1\_trpO}$-yEGFP-T_{ADH1}	This study
pCfB5397	CEN6/ARS4, pRS413U-*HIS3*, $P_{GAL1core\_3xtrpO}$-*yEGFP*-T_{ADH1}, P_{TEF1_trpO}-*mKate2*-T_{CYC1}	This study
pCfB5399	CEN6/ARS4, pRS413U-*HIS3*, $P_{GAL1core\_6xtrpO}$-*yEGFP*-T_{ADH1}, P_{TEF1_trpO}-*mKate2*-T_{CYC1}	This study
**Platform and library strain construction**		
pCfB176	CEN6/ARS4, pRS414-*TRP1*, P_{TEF1}-SpCas9-T_{CYC1}	DiCarlo *et al* [5]
pCfB4672	CEN6/ARS4, pRS413U-*HIS3*, P_{TEF2}-*mKate2*-T_{IDP1}, P_{GAL1}-*ACT1*-T_{ADH1}	This study
pCfB4673	CEN6/ARS4, pRS413U-*HIS3*, P_{TEF2}-*mKate2*-T_{IDP1}, P_{GAL1}-*CDC14*-T_{ADH1}	This study
pCfB9303	CEN6/ARS4, pRS415U-*LEU2*, P_{GAL1}-*ACT1*-T_{IDP1}	This study
pCfB9307	CEN6/ARS4, pRS415U-*LEU2*, P_{GAL1}-*ACT1*-T_{IDP1}, *TKL1-TAL1-PFK1-CDC19* (native expression cassettes)	This study
pCfB6842	2 *µ*, pESC-*LEU2,* P_{SNR52}-*ARO4*_gRNA-T_{SUP4}	This study
pCfB6843	2 *µ*, pESC-*LEU2,* P_{SNR52}-*ARO4*_gRNA-T_{SUP4}, P_{SNR52}-*TRP2*_gRNA_1-T_{SUP4}, P_{SNR52}-*TRP2*_gRNA_2-T_{SUP4}	This study
pCfB6844	2 *µ*, pESC-*LEU2,* P_{SNR52}-*TRP2*_gRNA_1-T_{SUP4}, P_{SNR52}-*TRP2*_gRNA_2-T_{SUP4}	This study
pCfB6903	2 *µ*, pESC-*LEU2,* P_{SNR52}-XI-2_gRNA-T_{SUP4}	This study
pCfB6904	2 *µ*, pESC-*LEU2,* P_{SNR52}-XI-3_gRNA-T_{SUP4}	This study
pCfB6909	2 *µ*, pESC-*LEU2*-P_{SNR52}-XII-5_gRNA-T_{SUP4}	This study
pCfB6916	2 *µ*, pESC-*URA3,* P_{SNR52}-XI-5_gRNA-T_{SUP4}	This study
pCfB6895	2 µ, pESC-*URA3*, P_{SNR52}-*PCK1*_gRNA-$T_{SUP4,}$ P_{SNR52}-*TAL1*_gRNA_1-$T_{SUP4,}$ P_{SNR52}-*TAL1*_gRNA_2-$T_{SUP4,}$ P_{SNR52}-*TKL1*_gRNA-T_{SUP4}	This study
pCfB9306	2 µ, pESC-*URA3*, P_{SNR52}-*pPFK1*_gRNA_1-$T_{SUP4,}$ P_{SNR52}-*pPFK1*_gRNA_2-$T_{SUP4,}$ P_{SNR52}-p*CDC19*_gRNA_1-$T_{SUP4,}$ P_{SNR52}-p*CDC19*_gRNA_2-T_{SUP4}	This study

Supplementary Table 8. Yeast strains engineered and used in study.

Name	Genotype	Reference
CEN.PK113-11C	*MAT***a** *his3*Δ1, *LEU2*, *ura3*-52, *TRP1 MAL2*-8c *SUC2*	EUROSCARF
CEN.PK2-1C	*MAT***a** *his3*Δ1, *leu2*-3_112, *ura3*-52, *trp1*-289, *MAL2*-8c *SUC2*	EUROSCARF
TrpA-1	*MAT***a** $P_{GAL1core\_6xtrpO}$-*yEGFP*-T_{ADH1}, P_{TEF1_trpO}-*mKate2*-T_{CYC1}, pCfB176	This study
TrpA-2	*MAT***a** $P_{GAL1core\_6xtrpO}$-*yEGFP*-T_{ADH1}, P_{TEF1_trpO}-mKate2-T_{CYC1}, *ARO4*^{wt}::*ARO4*^{K229L}, pCfB176	This study
TrpA-3	*MAT***a** $P_{GAL1core\_6xtrpO}$-*yEGFP*-T_{ADH1}, P_{TEF1_trpO}-mKate2-T_{CYC1}, *TRP2*^{wt}::*TRP2*$^{S65R, S76L}$, pCfB176	This study
TrpA-4	*MAT***a** $P_{GAL1core\_6xtrpO}$-*yEGFP*-T_{ADH1}, P_{TEF1_trpO}-mKate2-T_{CYC1}, *ARO4*^{wt}::*ARO4*^{K229L}, *TRP2*^{wt}::*TRP2*$^{S65R, S76L}$, pCfB176	This study
TrpNA-W	*MAT***a** *tkl1*Δ *tal1*Δ *pck1*Δ, P_{PFK1}::P_{REV1}-*PFK1*, P_{CDC19}::P_{RNR2}-*CDC19*, P_{PFK1}-*GAL4*$_{ad}$-*trpR*-T_{ADH1}, $P_{GAL1core\_3xtrpO}$-*yEGFP*-T_{ADH1}, P_{TEF1_trpO}-*mKate2*-T_{CYC1}, P_{PGK1}-*ARO4*^{K229L}-T_{ADH1}, P_{TEF1}-*TRP2*$^{S65R, S76L}$-T_{CYC1}, pCfB176, pCfB9307	This study

Supplementary References

1. Saitou, N. & Nei, M. The neighbor-joining method: a new method for reconstructing phylogenetic trees. *Mol. Biol. Evol.* **4**, 406–425 (1987).
2. Studier, J. A. & Keppler, K. J. A note on the neighbor-joining algorithm of Saitou and Nei. *Mol. Biol. Evol.* **5**, 729–731 (1988).
3. Jensen, N. B. *et al*. EasyClone: method for iterative chromosomal integration of multiple genes in Saccharomyces cerevisiae. *FEMS Yeast Res.* **14**, 238–248 (2014).
4. Zhang, J. *et al*. Engineering an NADPH/$NADP^+$ redox biosensor in yeast. *ACS Synth. Biol.* **5**, 1546–1556 (2016).
5. DiCarlo, J.E. *et al.* Genome engineering in Saccharomyces cerevisiae using CRISPR-Cas systems. *Nucleic Acids Res.* **41**, 4336-43 (2013).
