# Supplementary material for: Combining mechanistic and machine learning models for predictive engineering and optimization of tryptophan metabolism
Source: Nat Commun. 2020 Sep 25;11:4880. doi: 10.1038/s41467-020-17910-1 (PMC7519671; doi:10.1038/s41467-020-17910-1)
Supplement: Supplementary file 4 — Description of Additional Supplementary Files [file 41467_2020_17910_MOESM4_ESM.pdf]

Description of Additional Supplementary Files

File name: Supplementary Data 1

Description: Gene scores of genome-scale modelled (FBA) genes. Gene scores of all 192 genome-scale modelled (FBA) genes with significant changes in flux towards tryptophan production under glucose and ethanol conditions. A score higher than one means the gene is an up-regulation candidate, a score between zero and one means the gene is a down-regulation candidate, a score equal to zero means the gene is a knockout candidate, and a blank score means the gene is associated to reactions that do not change significantly in flux as tryptophan production increases under that particular condition. The four out of five gene targets identified by FBA and selected for this study are marked in bold. Related to Figure 1.

File name: Supplementary Data 2

Description: FBA results for metabolic all pathways. FBA results for all pathways in metabolism, including the number of gene targets predicted in each pathway, the total size of each pathway, the fraction of genes in each pathway that are gene targets, and the significance of that representation in each pathway compared to the rest of metabolism ("Whole metabolism"), indicated by a P-value computed with a two-sided Fisher's exact test. General pathways such as "carbon metabolism" and "biosynthesis of amino acids" were filtered out of the analysis.

File name: Supplementary Data 3

Description: Summary of promoter combinations, GFP synthesis rates, and growth rates. Promoter combination, mean specific rate of GFP synthesis, and growth rate (mean and standard error; n = 3) for all strains characterized in this study. The strains are ordered by decreasing the specific rate of GFP synthesis. The size of the color bars indicates promoter expression strength (see Figure 1), specific rate of GFP synthesis or growth rate. Line names containing numbers greater than 576 represent recommended strains, numbers from 481 to 576 control strains, and numbers below 481 library strains.

File name: Supplementary Data 4

Description: Primers used in study. Sequence features of interest are separated by a space.
