# Supplementary material for: Combining mechanistic and machine learning models for predictive engineering and optimization of tryptophan metabolism
Source: Nat Commun. 2020 Sep 25;11:4880. doi: 10.1038/s41467-020-17910-1 (PMC7519671; doi:10.1038/s41467-020-17910-1)
Supplement: Supplementary file 2 — Peer Review File [file 41467_2020_17910_MOESM2_ESM.pdf]

Reviewers' comments:

Reviewer #1 (Remarks to the Author):

This is a good paper that entails quite a substantial amount of work. The aim of the work is to find out the best promoter combinations (for 5 genes) maximizing tryptophan production in yeast. To achieve this aim, the paper presents i) a good span of promoter strength to sample from ii) a cleaver method to quickly build large combinatorial libraries, iii) a biosensor that is operational in the needed sensitivity range (1 to 100 mg/L) with a good correlation coefficient (concentration vs. fluorescence level), and iv) reasonable machine learning performances to find best promoter combinations (albeit not to predict accurately GFP synthesis rate). For all the above reasons, the paper deserves to be published after the authors address the following points.

General comment

In the abstract, introduction and discussion the reader is left to believe that very large datasets were used to train machine learning algorithms ("biosensor which enabled the sampling of >144,000 GFP intensity measurements" -- "1,728 isoclonal designs in a high-throughput") while at the end the two ML algorithms were trained on about 250 data points. The authors should make this clear as after all 250 is quite large when we are considering this number represents different engineered strains.

Minor comments

Title: Mechanistic models are used just to retrieve ⅘ gene used in the rest of the manuscript. The predictive engineering is mainly carried out by machine learning, I am therefore not sure mechanistic model deserves to be in the title.

Page 3-line 105. 'intelligently designed condensed libraries' could the authors expand a bit on the mathematical technique used to make this intelligent design is it statistically-based? block design based?

Page 3. Be careful using the terminology 'global optimum' as there is no guarantee machine learning or other stochastic techniques ever reach the global optimum.

Page 4 - Line 119: The 144000 data points are quite confusing knowing that the combinatorial space is 7776.

Figure 1. Fonts and drawing are rather small which makes it difficult to read.

Page 8 – line 259. Where do the 507 colonies come from?

Page 9 – line 274. "since there is no algorithm which is optimal for all learning tasks, we used two different learning approaches", it is unclear what are the learning tasks considering the only task asked to machine learning is to "predict promoter combinations expected to improve tryptophan productivity". Additionally, the two methods ART and EVOLVE take the same input and both predict GFP sensitive rate. The author should further clarify what they mean by different learning tasks.

Page 9. The rationale for choosing the two methods ART and EVOLVE is not well explained and justified.

Page 9. I assume the size of the data set used for training is about 250 as this is apparent from Figure

4. This should be clearly stated in the manuscript.

Page 9. Reading the method section, ART and EVOLVE predict GFP synthesis rate, therefore both models perform a regression this should be stated in the main text on page 9.

Figure 4. While dataset size increases, MAE on the test set decreases (for both methods) this is a good thing. However, the regression coefficients (obtained when trained on the whole set I suppose) are not that convincing. Looking at Figure 2e-f, I would argue that the models have no predictive value to estimate GFP synthesis rate. Could the authors comment on that, as this is not discussed at all in the main text?

Page 12 - line 407. The statement "Ultimately, in our case study, machine learning models have demonstrated significant predictive power" this does not seem right as far as GFP synthesis rate is concerned (Figure 4 d-e)

Page 22. Why was one-hot-encoding used to describe promoters with ART? Seems to me that numbers representing the different promoter strength would have been sufficient. Was one-hot-encoding also used with EVOLVE?

Page 22. Both ART and EVOLVE predict GFP synthesis rate but with different methods. ART is used for 'exploitation' to select 30 strains that have the highest GFP synthesis rate, while EVOLVE is used for 'exploration' to select 30 strains having the best-expected improvement (combination GFP synthesis rate and the uncertainty of prediction). It is not clear to me why EVOLVE could not be used for exploitation (removing uncertainty of prediction) and ART for exploration (adding uncertainty of prediction). Ultimately this raises the question of why two methods were used. Mixing exploitation and exploration is a standard strategy in active/reinforcement learning and is generally carried out by the same (ensemble of) method(s) but not separated ones.

Having said that, using two different methods is valuable and should not be discarded. What could have been done instead is to use ART and EVOLVE each for exploration and exploitation (2 exploration + 2 exploitation searches), and compare and merged the 4 searches to make recommendations for additional measurements.

Reviewer #2 (Remarks to the Author):

In this manuscript, the authors take on the challenge to introduce machine-learning into the world of microbial biotechnology. For this purpose, optimization of the Trp-production with S. cerevisiae was selected. Initially, the authors identified five known targets from mechanistic genome-scale model simulations and designed and constructed a large combinatorial library in which the promoter was always varied. Subsequently, heaps of data were collected (Trp-biosensor output, growth, sequencing data) to fed two different machine learning algorithms. These models predicted strain variants, which were significantly improved compared to the best published strain and the best strain design used to train the models.
The overall topic is very interesting – machine learning as the logical next step in strain design is definitely sth. (metabolic) engineers have to look into. However, considering the immense workload to generate the data to train the models and the rather limited outcome in terms of improvement presented as part of this study (at this development stage) one has to ask: How much more data is needed to make the algorithms even better? This is definitely an aspect, which has to be more discussed.

In the last years, adaptive laboratory evolution (ALE) made a huge comeback. I would agree that such an ALE-strategy is sometimes difficult to develop for improved product formation, but it is much less laborious and takes also completely unknown beneficial connections on the metabolic- and/or regulatory level of the microbial metabolism into account. It would be interesting if the authors discuss this old technology in the light of their results/approach (genome-scale-models/machine learning).
In general, the manuscript is well written, but appears to be a bit too long. Some aspects do not appear to be too important for the overall content, such as the lengthy description of the one-pot construction of the combinatorial library. This can be significantly shortened (or partly transferred to the suppl. information).

Reviewer #3 (Remarks to the Author):

The paper by Zhang et al describes the development of a baker's yeast strain for the production of tryptophan. While aromatics production in yeast using metabolic engineering has a long history, the approach presented here is new. It uses a combination of genome scale modelling for the prediction of improved strain designs, a high throughput approach to implement these designs with a large variety of expression strength and two machine learning approaches to use the phenotypic data from the different design, to predict the ideal designs.

The paper is certainly highly relevant and interesting. Before publication I would recommend the following improvements:

The paper is all about production, yet it does not provide a quantitative dataset. What are the yields, titers and rates of tryptophan production? Only percentages are given, but these are used in a confusing way. In the abstract it is stated that 106% improvement of accumulation is achieved (does this mean concentration?), later in line 350 it is given as 106% increase in rate but what are the absolute rates? Why are titers not given for the designs in comparison to the reference strain? How are the rates calculated? Specific? Volumetric? What was the biomass density etc. Here the paper is far too vague. One can only take the 106% as face value, but what does it really mean? Acknowledging that no proper fermentation was conducted in a bioreactor and so a carbon balance is not available, could the results be put in perspective of currently achieved yields, rates or titers for aromatics? https://doi.org/10.3389/fbioe.2018.00032.

Discussion of the best designs should be put into context of literature more thoroughly. E.g. Knockout of PYK was described as an optimization strategy in an earlier work on aromatics production in yeast https://doi.org/10.1016/j.ymben.2015.03.008

Materials and Methods are too brief in some sections: E.g. Line 672: ''Supernatants were separated….'' But how?

Minor comments:

References: Check format. Organisms in italics, page 28 shows some boxes lines 951-952
The reference (Radivojević et al) provided for the ART algorithm is not a peer-reviewed article. Please update if possible.

Line 341: For(?) construction….
Line 636: The success … was….
Line 595: Check ref Jessop

Resolution of figures in supplementary should be improved

Reviewer #4 (Remarks to the Author):

This manuscript first used a gnome-scale model (GSM) to predict 5 genes related to tryptophan synthesis in yeast. And then, with the help of CRISPR/Cas9 genome engineering tools, 6 promoters with different strength were used to drive these genes to build a combinatorial library with 7,776 (65) members. To facilitate high-throughput test of the library, a tryptophan biosensor was constructed with a dynamic range of 5 folds, and an operational range of ~2-200 mg/L trp. With the genotypic combinatorial library and phenotypic testing tool in hand, 507 colonies were tested separately, with output of >144,000 data points. These data were then used to predict the combination with optimized trp productivity; here commonly used ART model and EVOLVE algorithm were used. The new recommended designs improving tryptophan production by up to 17% compared to the best designs used for algorithm training. Combining GSMs with machine learning is certainly a promising approach and future direction of engineering cellular metabolism. This work is thus of certain interest. However, the significance of results and findings from this work is quite limited for the specific case of trp biosynthesis.

There are some shortcomings and unclear points in this manuscript:

1. Data size. As stated in the manuscript: "Following transformation, we randomly sampled 480 colonies from the library, together with 27 colonies from the five control strains (507 in total), and successfully cured 423", considering the repeated genotype (3.7% repeat showed Fig. 2B), only ~400 gonotype-phnotype association data at different cultivation time were acquired. The claim that >144,000 data points, 1,728 isoclonal designs were used is misleading. The authors should state this point more clearly.

2. Description. Page 9, lines 283-286. The sentence "As the quality ….." is not clear or ill-structured.

3. Prediction power. Although "an order of magnitude higher number of strains than in previous machine learning-guided metabolic engineering studies (Alonso-Gutierrez et al., 2015; Lee et al., 2013a; Redding-Johanson et al., 2011; Zhou et al., 359 2018a)", the prediction power seems to be not very good. Why? The quality of training dataset? Could iterative prediction strategy by feeding the predicted date to the initial library work? Could published models used in previous study yield better results? The authors should address these points or give comments on them.

4. Knowledge learned from the data? Which combinations give better productivity? Are there any rules or explanation behind it? Could these data help to optimize the genome scale model?

5. The conclusion cannot be fully supported by the data, e.g. "ultimately producing a total increase of 106% in tryptophan accumulation compared to optimized reference designs", they even did not give the data of "real" tryptophan concentration, only GFP fluorescence intensity. This problem seems to be severe when considering the strong scattering of the seemingly linear relationship between fluorescence intensity and extracellular tryptophan concentration showed in Fig. 3D. Furthermore, the Trp concentration (a few mg/L) achieved is very low and the improvement is marginal.

6. Novelty of the findings. In fact, the genotypes (knock-downs of both CDC19 and PFK1, low expression of TKL1 and high expression of TAL1) of the best performing strains (SP606, SP616)

predicted by machine-learning are more or less known in the literature or can be relatively easily inferred from the pathways by considering the fact that PEP and E4P are two important precursors of trp synthesis.

7. Target genes. It should be mentioned that in addition to PEP and E4P several other metabolites like glutamine, serine and 5-P-D-ribose-diphospahe are direct or indict precursors of trp synthesis. Genes related to the formation of these metabolites should be also considered. The real challenge for efficient trp synthesis is dynamically balancing the synthesis of these precursors depending on growth rate, the desired trp yield and productivity. The export of trp is also very crucial. The GSMs and machine leaning algorithms cover only a small part of a very complex metabolism and the regulations (many!) are not considered at all. Some more recent advanced studies on the metabolic engineering of trp pathway should be considered. Very high titer, yield and productivity of trp have been achieved by rational metabolic engineering.

8. Biosensor. The existence of biosensor in the cell will have influences on tryptophan production, it would be better to test the tryptophan production without the biosensor plasmid.

Response to Reviewers' comments

We thank the editor and the reviewers for the interest in our manuscript, for the generally positive remarks, and also for the constructive criticism raised. In our revised manuscript we have included new experiments related to tryptophan measurements and new computational analyses as requested by the reviewers. Based on this we have substantially edited manuscript text, improved readability of figures, and expanded supplementary information to include more quantitative data according to the valuable comments raised.

In the resubmission, we have included both "clean" and "tracked changes" revised manuscript files. We hope the edits, all marked in green, and our detailed point-by-point responses to all comments raised, all marked in red, will be satisfactory to the editor and reviewers, and we look forward to your feedback.

Reviewer #1 (Remarks to the Author):

This is a good paper that entails quite a substantial amount of work. The aim of the work is to find out the best promoter combinations (for 5 genes) maximizing tryptophan production in yeast. To achieve this aim, the paper presents i) a good span of promoter strength to sample from ii) a clever method to quickly build large combinatorial libraries, iii) a biosensor that is operational in the needed sensitivity range (1 to 100 mg/L) with a good correlation coefficient (concentration vs. fluorescence level), and iv) reasonable machine learning performances to find best promoter combinations (albeit not to predict accurately GFP synthesis rate). For all the above reasons, the paper deserves to be published after the authors address the following points.

>> We thank the reviewer for the positive remarks on our study.

General comment

1. In the abstract, introduction and discussion the reader is left to believe that very large datasets were used to train machine learning algorithms ("biosensor which enabled the sampling of >144,000 GFP intensity measurements" -- "1,728 isoclonal designs in a high-throughput") while at the end the two ML algorithms were trained on about 250 data points. The authors should make this clear as after all 250 is quite large when we are considering this number represents different engineered strains.

>> This is a valid point. We see that clarity on the numbers used for generating the training data set can be improved in the manuscript text. While the entire list of data points obtained in this study equalled >124,000 (507 strains + 3 replicates + 82 time points = 124,722, see also Figures S4-S5), we do agree with the reviewer that the actual number used for training our models could be made clearer in the Introduction and Discussion. In the revised manuscript we have now made the following clarifications:

Abstract:
The approach harnesses efficient one-pot library construction and high-throughput biosensor-enabled screening for successful forward engineering of complex aromatic amino acid metabolism in yeast, with the best machine learning-guided design recommendations improving tryptophan titer and productivity by up to 74% and 43%, respectively, compared to the best

designs used for algorithm training~~, and ultimately producing a total increase of 106% in tryptophan accumulation compared to optimized reference designs.~~ Thus,...

Introduction:
In order to train predictive models for ~~high~~ tryptophan biosynthesis rate in yeast, we collected >124,000 experimental time-series data points derived from fluorescent read-outs of a newly engineered tryptophan biosensor encoded into >500 different strain designs. This enabled selection of optimal sampling time-points, from which we explored fluorescence synthesis rates of approximately 3% (250/7,776) of the possible genetic designs...

and

Discussion:
To gather the large high-quality data set required for machine learning approaches, we developed a biosensor which enabled the sampling of >124,000 GFP intensity measurements (82 time points) as a proxy for tryptophan flux for 1,521 isoclonal designs (3 replicates x 507 strains) in a high-throughput fashion, of which data from 250 strains were eventually used for successful training of ML algorithms (Figures 3E, S5A).

Also, we wish to emphasise that the full data filtering outline is presented in the Figure S5 as already referred to, and that all data and accompanied data analysis can be found directly in the github repository associated with this study. We are sorry about the lack of clarity in the original manuscript, yet we hope these clarifications satisfy the reviewer.

Minor comments

2. Title: Mechanistic models are used just to retrieve ⅘ gene used in the rest of the manuscript. The predictive engineering is mainly carried out by machine learning, I am therefore not sure mechanistic model deserves to be in the title.

>> While we do understand the immediate concern of the reviewer, we wish to emphasize that the mechanistic model was crucial to pinpoint which reactions to apply the ML approach to. More specifically, the prior biological knowledge encoded in the genome-scale model was critical to obtain these results. Moreover, we wish to emphasize that while the genome-scale model ranked the impact of single biochemical reactions, we eventually combined the single gene targets, into one combinatorial library from which >50% of the designs had higher tryptophan biosynthesis rate than the reference strain (Figs. 3E and 4E-F). This “hit rate” would not have been observed if combining randomly sampled gene targets.

Based on this response, we hope to have convinced the reviewer that “Predictive” indeed goes for both the mechanistic modelling and the mathematical modelling.

3. Page 3-line 105. ‘intelligently designed condensed libraries’ could the authors expand a bit on the mathematical technique used to make this intelligent design is it statistically-based? block design based?

>> Valid point. In order to clarify this term we have made the following clarification to the manuscript:

Introduction:

However, this challenge can be mitigated by the use of intelligently designed condensed libraries which allow uniform discretisation of multidimensional spaces: e.g. by using well-characterized sets of DNA elements controlling the expression of candidate genes at defined levels as opposed to using more less-/non-characterized random elements[29,30].

In this study we restricted us to using a relatively few well characterized promoters that span the space of promoter strengths from weak to strong. Thereby making a uniform discretisation of the space of promoter strengths which we consider to be a condensed library (as the term is used in Jeschek et al. 2016 and 2017), compared with using non-characterized promoters. If using random non-characterised we would need to test many more promoters to investigate the same space of promoter strengths. The library could indeed have been compressed further, i.e. by using e.g. statistically-based or block design based techniques as suggested by the reviewer, and this could indeed be relevant to investigate in the future. However, such compression would require using another method for strain library construction than the one pot transformation procedure used in this paper, for which we cannot enforce restrictions on specific part combinations.

4. Page 3. Be careful using the terminology ‘global optimum’ as there is no guarantee machine learning or other stochastic techniques ever reach the global optimum.

   >> Correct, and a very relevant point to highlight. Throughout the manuscript we have tried to highlight that searching (not identifying!) for global optima needs critical attention to both the library parameters of relevance and the efficiency of library construction in order to combat combinatorial explosions. Still, even when this is taken into consideration there is indeed no guarantee that mathematical models will ever identify global optima. We have now clarified this in the revised manuscript.

   Introduction (second-last paragraph):
   Still it should be noted, that even with intelligent choice of design parameters and efficient library construction, there is no guarantee mathematical models will reach such a global optimum.

5. Page 4 - Line 119: The 144000 data points are quite confusing knowing that the combinatorial space is 7776.

   >> We are sorry about the confusion and deeply regret both the typo (124,722 not >144,000 - derived from mistaken calculation of data points from triplicates of all 576 strain designs, and not the actual 507 strains analysed following filtering), as well as not having clarified the calculations behind the number well enough. Based on the response to this comment, and to comment #1 (see above) we hope this has now been clarified. See also Fig. S5 for further clarification.

6. Figure 1. Fonts and drawing are rather small which makes it difficult to read.

   >> The size of fonts and drawings have now been increased. Minimum font is now 8 pt (6 pt before). Also, several figures have been enlarged 25% to further ease readability. We hope this resolves the difficult reading.

7. Page 8 – line 259. Where do the 507 colonies come from?

>> As indicated in the Result part (section “One-pot construction of the combinatorial library” and Figure S5), the 507 colonies arise from 480 randomly picked colonies plus 27 control strains. However, to further clarify the underlying filtering and calculations related to the numbers stated we have further clarified this in the revised manuscript:

Results (section Engineering a tryptophan biosensor for high-throughput library characterization):
To do so, we measured time-series data of OD and GFP at 82 time-points in triplicates for all 507 colonies (that is 480 from the library and 27 from the control strains), covering a total of 124,722 data points (Figures S4-S5).

and in,

Discussion:
...the sampling of >124,000 GFP intensity measurements (82 time points) as a proxy for tryptophan flux for 1,521 isoclonal designs (3 replicates x 507 strains) in a high-throughput fashion, of which data from 250 strains were eventually used for successful training of ML algorithms (Figures 3E, S5A).

8. Page 9 – line 274. “since there is no algorithm which is optimal for all learning tasks, we used two different learning approaches”, it is unclear what are the learning tasks considering the only task asked to machine learning is to “predict promoter combinations expected to improve tryptophan productivity”. Additionally, the two methods ART and EVOLVE take the same input and both predict GFP sensitive rate. The author should further clarify what they mean by different learning tasks.

   >> Relevant point. What we meant to say is that the well known “No Free Lunch”-theorem indicates that there is no algorithm that is best for all conceivable general learning tasks. Hence, the standard approach is to try a variety of different algorithms (e.g. Orlenko et al., 2019, PMID: 31702773; Costello & Martin, 2018, PMID: 29872542). Here, we do so by trying two different learning approaches: ART and EVOLVE - yet these algorithms had indeed only one learning task (i.e. to predict promoter combinations). We have now included this clarification:

   Result (section “Using machine learning to predict metabolic pathway designs”):
   Since there is no single algorithm which is optimal for all conceivable general learning tasks[62], we decided to improve our chances by using two different machine learning approaches for the single regression learning task of predicting promoter combinations controlling five genes that best improve GFP biosynthesis rates, as a proxy for tryptophan productivity: the Automated Recommendation Tool (ART) and EVOLVE algorithm[63,64] (see also METHODS).

9. Page 9. The rationale for choosing the two methods ART and EVOLVE is not well explained and justified.

   >> While the major description on the differences in modelling parameters of ART and EVOLVE can be found in the Methods (section “Modelling”), we apologize for the brevity in explaining the rationale for choosing ART and EVOLVE when first mentioned in the Result part page 9. As stated before, there is no single approach that works best for every single task (see our reply to comment #8). Hence, we tested two different approaches, making use of different

ensemble regressors and modelling parameters (e.g. outlier calling, uncertainty level), and compared their learning curves, recommendations and predictive power. In line with another comment relating to this Result section (see comments #11 and #15-16 below), we have now specified the section on page 9 and provided a link to the Methods section as already indicated in our reply to comment #8.
Furthermore, we wish to highlight that the demonstration of this comparative study should be of value to the general readership, and something we also return to in the Discussion:

Discussion:

With this in mind, a relevant guideline for choosing a recommendation approach should focus on the desired outcome: the explorative approach providing a more diverse set of recommendations (Figure 4C-D), whereas the exploitative approach provides less varied recommendations. We observed the largest improvement in titer and productivity when using the exploitative approach (Figure 4E-F, Figure S7). However, if subsequent design-build-test-learn cycles are performed, the diversity of recommendations of the explorative approach could help avoid local optima of tryptophan production (Figure 4E-F).

10. Page 9. I assume the size of the data set used for training is about 250 as this is apparent from Figure 4. This should be clearly stated in the manuscript.

>> Good point, and in line with comments #1 and #7. We have included the following text in the manuscript in order to clarify how we reached the 250 strains used for training:

Result (section "Using machine learning to predict metabolic pathway designs")
Following this, approximately 58% (266/461) of the growing strains remained after filtering, while another 3% of the remaining data was removed because of lack of reproducibility (high error in triplicate measurements), ultimately leaving high-quality sequencing and GFP data from 250 genotypes as input training data set (Figure S5)*.*

11. Page 9. Reading the method section, ART and EVOLVE predict GFP synthesis rate, therefore both models perform a regression this should be stated in the main text on page 9.

>> We agree, and this has now been clarified. Please see our revised text from our response to comment #9.

12. Figure 4. While dataset size increases, MAE on the test set decreases (for both methods) this is a good thing. However, the regression coefficients (obtained when trained on the whole set I suppose) are not that convincing. Looking at Figure 2e-f, I would argue that the models have no predictive value to estimate GFP synthesis rate. Could the authors comment on that, as this is not discussed at all in the main text?

>> We thank the reviewer for acknowledging the learning curves presented in Figure 4A-B. The R^2 values are indeed calculated for all the points shown (not only grey, this is now stated in the legend to Figure 4E-F). For the results presented in Figure 4E-F (we assume this is what the reviewer refers to), we consider the models to obtain very respectable values (R^2 = 0.60 and 0.44, grey dots) in making cross-validated predictions (i.e. for data the model has NOT seen, and not predictions using whole training data set) for biological systems, which are notoriously difficult to predict. We acknowledge that, for both approaches, the recommended strains' productivity (blue dots) was poorly predicted, likely because it involved an extrapolation effort which is a known weakness for machine learning methods (as stated in the main text).

However, it is also true that despite this lack of extrapolation power, ART and EVOLVE were able to predict promoter combinations that improve production compared to the base strain in 35/39 cases. This shows that accurate predictive models are not absolutely required to provide effective recommendations.

In order to discuss these findings in more detail in the main text, we have now discussed this further in the manuscript.

Results (Machine learning-guided engineering of designs with high tryptophan productivity):
….and the explorative approach included recommendations based on a more diverse set of promoters than the exploitative approach (Figure 4C-D). Aligned with this, we observed that the recommendations from the EVOLVE approach also included a fraction of combinatorial designs with GFP synthesis rates below the reference strain (Figure 4F). Still, taken together, when run in parallel, ART and EVOLVE approaches successfully enable predictive engineering of tryptophan biosynthesis strain designs, and for both approaches even strains with tryptophan biosynthesis rates beyond those previously observed for training the models (Figure 4E-F, Table S10-S11).

Figure 4 Legend:
Data is shown for library and control strains (grey markers; green markers show the platform strain expressing ARO4^{K229L} and TRP2^{S65R,S76L}), as well as for recommended strains (blue markers; orange markers show recommendations that overlap between the two approaches). R-squared values are for cross-validated predictions for the whole data set (not only training set data).

13. Page 12 - line 407. The statement "Ultimately, in our case study, machine learning models have demonstrated significant predictive power" this does not seem right as far as GFP synthesis rate is concerned (Figure 4 d-e)

>> This point is related to comment #12. While we do not agree that the models have no predictive power judged from Figure 4E-F, we have now provided a more detailed statement to the cited sentence:

Discussion:
Ultimately, in our case study, machine learning models have demonstrated good performance in predicting GFP biosynthesis rates for the training data designs (grey dots in Figure 4E-F), while the recommended strains' biosynthesis rates were less accurately predicted, likely because it involved an extrapolation effort which is a known weakness for machine learning methods (blue dots in Figure 4E-F).

14. Page 22. Why was one-hot-encoding used to describe promoters with ART? Seems to me that numbers representing the different promoter strength would have been sufficient. Was one-hot-encoding also used with EVOLVE?

>> Good point. We tried both approaches (one-hot-encoding and numbers representing promoter strength) and the one-hot-encoding approach produced slightly better results in terms of the MAE and R^2. For clarity we have added the following sentence to the manuscript.

Quantification and statistical analysis (Modelling):
For both approaches, we tried encoding the promoter variables both as numbers ordered

according to the counts from the RNAseq experiment (i.e. promoter strength[44]) and as one-hot-encoding, and chose the one-hot-encoding because it produced a lower MAE values and higher R-squared values.

15. Page 22. Both ART and EVOLVE predict GFP synthesis rate but with different methods. ART is used for 'exploitation' to select 30 strains that have the highest GFP synthesis rate, while EVOLVE is used for 'exploration' to select 30 strains having the best-expected improvement (combination GFP synthesis rate and the uncertainty of prediction). It is not clear to me why EVOLVE could not be used for exploitation (removing uncertainty of prediction) and ART for exploration (adding uncertainty of prediction). Ultimately this raises the question of why two methods were used. Mixing exploitation and exploration is a standard strategy in active/reinforcement learning and is generally carried out by the same (ensemble of) method(s) but not separated ones.

    >> The two methods were developed in different groups (one academic, one industrial), and in this collaboration the two complementary approaches stood out as interesting subjects to test for their ability to predict promoter combinations for tryptophan biosynthesis rates from a single design-build-test-learn cycle. Most importantly, the two methods make use of different ensemble regressors and modelling parameters (e.g. outlier calling, uncertainty level), and we wished to explore the impact of such differences on the predictive power of two methods based on a single data sampling covering many designs. It is evident that the basic strategies could have been mixed, as successfully conducted previously (e.g. in the multi-iterative approach by Borkowski *et al* (https://www.biorxiv.org/content/10.1101/751669v1.full.pdf), but in this project we were interested to learn the power of each approach trained on a large data set from a single iteration.

    Having said this, we think the comment is very relevant, and we have now run both methods (ART and EVOLVE) in both exploratory and exploitative modes and compared the results (see answer to comment #16)

16. Having said that, using two different methods is valuable and should not be discarded. What could have been done instead is to use ART and EVOLVE each for exploration and exploitation (2 exploration + 2 exploitation searches), and compare and merged the 4 searches to make recommendations for additional measurements.

    >> Relevant point indeed. We can see reviewer # 1's comment that there is a logic problem in not enforcing variable control, and thus making the approaches less straight-forward to compare. As suggested, we have therefore used both models to search for complementary recommendations (as also mentioned in comment #15); Explorative with ART and exploitative with EVOLVE. The results obtained are now presented in new Tables S10-S11 and Figures S8, and Discussion has been updated accordingly.

    Discussion:
    This could be used to argue that more engineering iterations on even smaller data sets, potentially coupled to mixed exploitation and exploration approaches as recently demonstrated for cell-free production[68], should be a valid avenue for ML-guided engineering of even less genetically-tractable chassis, and for which no high-throughput screening method may even exist. With regards to this, we performed a follow-up test running the ART and EVOLVE approaches in explorative and exploitative modes, respectively. Here, we observed that the recommendations from EVOLVE in exploitative mode had overlaps of 20% (6/30) and 23% (7/30) to ART recommendations in exploitative and explorative mode, respectively. Complementary to this, the recommendations from ART in explorative mode only had overlaps

of 3% (1/30) and 0% (0/30) to EVOLVE recommendations in exploitative and explorative mode, respectively (Figure S8), indicating that the uncertainty of prediction of high GFP synthesis rate weighted differently for the two models in explorative mode.

Furthermore,the necessary ART code has been added to the script and the results placed in Supplementary Information

Reviewer #2 (Remarks to the Author):

In this manuscript, the authors take on the challenge to introduce machine-learning into the world of microbial biotechnology. For this purpose, optimization of the Trp-production with S. cerevisiae was selected. Initially, the authors identified five known targets from mechanistic genome-scale model simulations and designed and constructed a large combinatorial library in which the promoter was always varied. Subsequently, heaps of data were collected (Trp-biosensor output, growth, sequencing data) to fed two different machine learning algorithms. These models predicted strain variants, which were significantly improved compared to the best published strain and the best strain design used to train the models.

1. The overall topic is very interesting – machine learning as the logical next step in strain design is definitely sth. (metabolic) engineers have to look into. However, considering the immense workload to generate the data to train the models and the rather limited outcome in terms of improvement presented as part of this study (at this development stage) one has to ask: How much more data is needed to make the algorithms even better? This is definitely an aspect, which has to be more discussed.

>> First we wish to thank the reviewer for highlighting the timeliness of, and level of general interest in, our work. We are pleased to see that the reviewer acknowledges that our work contributes to the early stages of next-generation ME using ML-guided strain design. We are asked to elaborate our discussion on how much more data is needed to further refine predictive models, especially considering the limited improvements observed from our study. While we think this study is an important first *stress-test* of ML-guided engineering from a single DBTL data generation cycle, it has been reported that doing several DBLT cycles increases the power of the approach (Radivojevic et al, 2019; (Borkowski et al.)). Also, more data would allow us to expand the number of reactions targeted (from the 5 chosen here), hence allowing for the exploration of more phase space and possibly achieving better results. As such, the amount of data matters. However, estimating how many more DBTL cycles are needed to improve predictions can only be extrapolated from performing ~5 DBTL cycles (Radivojevic et al, 2019), which was not done in this case (here we used 1 engineering cycle). Still, a major learning from this study is the need to focus on the trade-offs between data generation and the numbers of iterations allowed for, but also how to enable sampling of high-quality data - and not necessarily more data per DBTL cycle. In the revised manuscript we have now included a discussion on both data amount, quality, and engineering iterations.

Discussion:

Another critical aspect to discuss from this study, is the amount and quality of data needed in order to increase the impact (e.g. improving titers, rates and yields) and reduce model uncertainty. From this study, we argue that biosensors for time-resolved sensing of cellular metabolism not only enable sampling of large amounts of data points, but most importantly also facilitate the identification of a smaller sampling space for high-quality determination of metabolite biosynthesis rates (Figure 3E). Specifically, we initially sampled triplicate measurements for 82 time points for all 576 strains, which when compared to growth,

ultimately allowed us to select 15 time points of relevance for calculating maximal GFP biosynthesis rates. Likewise, while the one-pot library construction used in this study had an estimated coverage of 48% of the full combinatorial design space, the amount of strains used for training the algorithms only covered approx. 3%, yet enabled predictive engineering following a single design-build-test-learn cycle.

With respect to the comment on "the rather limited outcome in terms of improvement presented as part of this study (at this development stage)", we wish to emphasize that this case study only focused on a limited set of overrepresented metabolic pathways, and only on the impact of transcriptional regulation. Yet, we hope the reviewer will acknowledge that the framework put forward in this study should be easily scalable to genetic targets beyond the 5 genetic locations and promoter replacement used as edit types in this study. Now that we have identified optimal sampling time point for tryptophan biosynthesis rate, the logical next step would indeed be to do more iterations and on more types of edits (e.g. transport, more pathways)(see also comment #5 from Reviewer #4 on this subject).

2. In the last years, adaptive laboratory evolution (ALE) made a huge comeback. I would agree that such an ALE-strategy is sometimes difficult to develop for improved product formation, but it is much less laborious and takes also completely unknown beneficial connections on the metabolic- and/or regulatory level of the microbial metabolism into account. It would be interesting if the authors discuss this old technology in the light of their results/approach (genome-scale-models/machine learning).

   >> ALE is powerful technology for genome-wide evolution-guided optimization of user-defined traits, albeit, as also indicated by the reviewer, with relatively few successful examples to boost production (e.g. carotenoid production by Reyes et al., 2014; succinate production by Tokuyama et al., 2018). While, we wish to focus this study on the current possibility of using mechanistic and ML-guided models for predictive engineering of cellular metabolism as compared to identification of global changes of metabolism from non-intuitive adaptive events based on evolution, we agree with the reviewer on the context-relevance of ALE, and have now included a brief insert of this in the updated manuscript Introduction and first senstence of Discussion to highlight the complementarity of ALE vs the approach used in this study. Also, it must be noted that ML, unlike ALE, can be used to produce outcomes for which there is no selective pressure (e.g. matching a metabolite concentration for a desired beer taste profile, Radivojevic et al, 2019).

   Introduction:
   These promises leverage tools and technologies developed over recent decades which include both non-intuitive evolution-guided approaches, such as adaptive laboratory evolution [3,4], as well as rational approaches combining mechanistic metabolic modeling, targeted genome engineering, and robust bioprocess optimization; ultimately aiming for accurate and scalable predictions of cellular phenotypes from deduced genotypes[5–7]

   Discusssion:
   In this study we wished to focus on the current possibility of using mechanistic and ML-guided models for predictive engineering of cellular metabolism as compared to sequential trial-and-error metabolic engineering iterations, or based identification of global changes of metabolism from non-intuitive adaptive events based on evolution. From this, we have demonstrated that....

   In general, the manuscript is well written, but appears to be a bit too long. Some aspects do not appear to be too important for the overall content, such as the lengthy description of the one-pot

construction of the combinatorial library. This can be significantly shortened (or partly transferred to the suppl. information).

>> While we have taken the liberty to expand some aspects of the manuscript based on reviewers' recommendations, we have also now shortened the description of the creation of the platform strain and the one-pot library construction procedure in the main Result text.

Reviewer #3 (Remarks to the Author):

The paper by Zhang et al describes the development of a baker's yeast strain for the production of tryptophan. While aromatics production in yeast using metabolic engineering has a long history, the approach presented here is new. It uses a combination of genome-scale modelling for the prediction of improved strain designs, a high throughput approach to implement these designs with a large variety of expression strength and two machine learning approaches to use the phenotypic data from the different design, to predict the ideal designs.

The paper is certainly highly relevant and interesting. Before publication I would recommend the following improvements:

>> We thank the reviewer for acknowledging the novelty of our approach, and for finding our study relevant and interesting.

1. The paper is all about production, yet it does not provide a quantitative dataset. What are the yields, titers and rates of tryptophan production?

>> Important point. We do think that our manuscript in general encompasses a lot of quantitative data related to strain performance (e.g. Supplementary Figures S4, S5 as well as Supplementary Tables S5, S8, and S9). Additionally, all experimental data and all calculations regarding the determination of synthesis rates used for modelling can be found both in the Experimental Data Depot (EDD) hosted by JBEI and in the jupyter notebook available at https://github.com/sorpet/Zhang_and_Petersen_et_al_2019. Here, the calculations are made in python and the calculated values such as rate of GFP synthesis and growth rates are outputs from this script. Please also see text for calculations of GFP biosynthesis rates in text associated with Figure S4.

Having said this, to further enable the easy track of the gathered experimental data we have now included all specific rates of GFP synthesis and growth rates (mean and standard error; n = 3) for all strains characterized in this study in new Table S12. Last but not least, we have now also measured tryptophan titers for 7 representative strains spanning a large range of GFP biosynthesis rates (new Figure S7, see also response to comments #2 and #3 below).

In terms of rates, we have a limitation in our experimental setup in that the tryptophan biosynthesis rate is calculated from the GFP synthesis rate, which is affected by oxygen depleting before the growth stops (again, please find our text describing this in Figure S4). This means that we cannot accurately measure rate values from the entire growth period but only from the period with remaining oxygen. With that said, we have estimated the average tryptophan biosynthesis rate during the 24 hour cultivation (new Figure S7, panel C).

Furthermore, in addition to all other quantitative data already existing in our manuscript, we wish to emphasize that this manuscript is not "*all about production*". This manuscript is most importantly a first demonstration of a novel metabolic engineering approach combining mechanistic modelling, high-throughput screening, and mathematical modelling to infer predictive design of complex pathways, and lays the foundation to explore further the improvement of tryptophan biosynthesis rates, and potentially in the future to couple this to

metabolic sinks for head-to-head comparisons with prior art. Again, we are happy to see that the reviewer already acknowledged this in the Resume, and we hope the updated manuscript, including new experimental data, more readily accessible quantitative data and rate calculations now satisfies the reviewer.

2. Only percentages are given, but these are used in a confusing way. In the abstract it is stated that 106% improvement of accumulation is achieved (does this mean concentration?), later in line 350 it is given as 106% increase in rate but what are the absolute rates?

>> This is a relevant point to clarify. In Figure 3 we show that GFP levels from the TrpR-based biosensors offer a robust proxy for tryptophan. Extending from this, we report the specific tryptophan biosynthesis rates based on the GFP biosynthesis rates (see Supplementary Figure S4 + text), and then compare all measured biosensor-derived values of engineered strains to the GFP biosynthesis rate of the reference strain with native promoters for all 5 candidate genes and allosteric regulation de-sensitized (strain ID SP507 in new Table S12). As such the 106% improvement refers to >2-fold increase in GFP biosynthesis rate (i.e. productivity) of the best performing recommended strain design (SP606) compared to the reference strain (SP507).

Having said this, in the updated manuscript we now also specify improvements based on new measurements of absolute HPLC-derived tryptophan titers (Figure S7 - see also response to comment #1). Beyond the updated Table S12 and Figure S7, we have now also updated the Abstract, Introduction, Result and Discussion part based on the newly obtained tryptophan measurements.

We hope this takes away the confusion experienced by the reviewer.

Abstract:
..the best machine learning-guided design recommendations improving tryptophan titer and productivity by up to 74% and 43%, respectively, compared to the best designs used for algorithm training~~, and ultimately producing a total increase of 106% in tryptophan accumulation compared to optimized reference designs.~~

Introduction:
Predictive models based on these algorithms enabled construction of designs exhibiting up to 74% higher tryptophan titers ~~biosynthesis rates~~ and 43% higher tryptophan productivities ~~than the best strain design used for algorithm training a state-of-the-art high-tryptophan reference strain, and up to 17% higher rate~~ than best designs used for training the models.

Result (Machine learning-guided engineering of designs with high tryptophan productivity):
...with the best recommendation (SP606) having a measured GFP synthesis rate 106% higher than the already improved platform design (SP507), and 17% higher than the best one (SP271) in the library sample (Figure 4E-F). This has been confirmed by HPLC analysis from small-scale deep-well batch cultivations, where we observed the strain SP606 having a 74% and 43% improvement in tryptophan titer and productivity, respectively, compared to the best strain design from the library sample (SP271)(Figure S7).

Discusssion:
In total, we managed to increase tryptophan titers and productivity by up to 74% and 43%, respectively, compared to an already improved reference strain ($ARO4^{K229L}$ and $TRP2^{S65R, S76L}$).

3. Why are titers not given for the designs in comparison to the reference strain?

>> This is a valid point. We have now included tryptophan titers and estimated rates of selected library and recommended strain designs, and compared these to the reference strain (SP507)(Figure S7). Result part has been updated related to this. Please see our response to comment #1 and #2.

4. How are the rates calculated? Specific? Volumetric?

>> We are sorry this has not been made more clear in our manuscript. Indeed, the rates reported are specific rates. We originally placed our description of how the rates were calculated in connection with Figure S4 (on parameter estimation from time series data). To make this even clearer, this description is now also referenced directly in the Methods section:

Methods:
Specific GFP synthesis rates were calculated as the difference in GFP divided by the difference in time (MFI/h) in the OD_{600} interval from 0.075 to 0.150, as measured by a Synergy Mx Microplate Reader from BioTek (a detailed description of the rationale behind this method can be found in connection with Figure S4).

5. What was the biomass density etc. Here the paper is far too vague. One can only take the 106% as face value, but what does it really mean?

>> In this study we did not measure biomass for the library strains. However, as mentioned in our answer to comment #1 and #4, we have now included more quantitative data on growth rates and increased the visibility of the detailed description of the method for calculating GFP synthesis rates. Also, as mentioned in our response to comment #2, we hope the reviewer agrees with us that the addition of new measurements on absolute tryptophan titers, the biomass densities and calculated productivities (Figure S7) enables easier comparison with prior art (see also updated Discussion), and improves the overall value of our study.

6. Acknowledging that no proper fermentation was conducted in a bioreactor and so a carbon balance is not available, could the results be put in perspective of currently achieved yields, rates or titers for aromatics? https://doi.org/10.3389/fbioe.2018.00032.

>> Thanks for raising an interesting point and an important reference. While it will not be fair to make a head-to-head comparison between the titers and rates obtained from this study (e.g. 96-well small-scale batch, low oxygen) and prior art, we have now included a section in our revised Discussion of the manuscript highlighting previous studies on bioprocess optimization and metabolic engineering for tryptophan and tryptophan-derived products. This could enable further improvements of the best-performing strain designs identified in this study based on machine-learning.

Discussion:
While discovery of strain designs with titers and rates outcompeting previously reported high-aromatics producers was not the main motivation for the study, it should be mentioned that all strains tested in this study produce much lower mg/L levels of tryptophan compared to previous studies focusing on metabolic engineering and bioprocess optimization for aromatics overproduction (Figure 3D, Figure S7)[33]. Indeed, as a suggestion for further optimization, it is possible that the reference strain used in this study is still subject to certain levels of feedback inhibition, as suggested by recent studies for aromatic amino acids derivatives[69,70]. Furthermore, the use of fed-batch cultivations as part of a bioprocess optimization would also be expected to

enable cells to accumulate higher tryptophan titers compared to the titers obtained based on short batch cultivations in 96-well deep plates with low oxygen levels used in this study.

7. Discussion of the best designs should be put into context of literature more thoroughly. E.g. Knockout of PYK was described as an optimization strategy in an earlier work on aromatics production in yeasthttps://doi.org/10.1016/j.ymben.2015.03.008

>> Thanks for the suggestion. In addition to the updated Discussion based on comment #6, we have further updated the revised Discussion to include more references to seminal work on aromatics production in microbes.

Discusssion:
Despite the low production, there is still a positive correlation between tryptophan titer/productivity and the GFP synthesis rate (Figure 3C-D, Figure S7), and the large-scale data set from this study provides examples of results anticipated based on rational engineering as well as non-intuitive predictions, enabling further advancement of the biological understanding of tryptophan metabolism. For instance, the best performing strain (SP606, Tables S8 and S12) predicted by machine-learning, included knock-downs of both CDC19 and PFK1, corroborating our intuitive strategies for increasing precursor availability: i.e. lower pyruvate kinase activity would lead to higher PEP pools, while limiting glycolysis redirects carbon flux into PPP and subsequently increases E4P[36]. Indeed, in bacteria, pyruvate kinase knockout has been used for the overproduction of shikimate-pathway derived aromatics products in bacteria[71–73]. Likewise, since yeast cells with CDC19 deletion cannot grow on glucose[74], dynamic silencing of CDC19 and PYK2 have been used for boosting production of para-hydroxybenzoic acid (PHBA)[75], just as expression of a mutant CDC19 pyruvate kinase with seemingly lower activity, in combination with overexpression of transketolase (TKL1), have been demonstrated to improve 2-phenylethanol (2PE) production in yeast[76]. On the contrary, a similar strategy with lower CDC19 activity, but in combination with zwf1Δ deletion (lacking the committed step towards the oxidative branch of PPP) was shown to reduce tyrosine titers[77]. Surprisingly, the top-5 strains predicted to have high tryptophan biosynthesis rates (SP606, SP616, SP624, SP588 & SPSP620, Tabel S8) all had low expression of TKL1...

8. Materials and Methods are too brief in some sections: E.g. Line 672: ''Supernatants were separated….'' But how?

>> Point taken. We have included more details in the Methods sections now, including the following clarification:

Methods (*Validation of biosensor by HPLC*):
Supernatants of cultivated strains were separated from the culture broth using AcroPrep Advance 96-Well Filter Plates (Pall Corporation) and centrifugation (5 min at 4000 rpm) following 24 hrs of cultivation in synthetic dropout medium without tryptophan and histidine.

Furthermore, we have moved detailed description of the construction of the reference strain and the library into the Methods section (see also suggestion put forward by Reviewer #2, comment #2).

Methods (Platform strain construction):

As CDC19 is an essential gene, and deletion of PFK1 causes growth retardation[48,49], this genetic background was deemed unsuitable for efficient one-pot transformation. For this reason our platform strain for library construction had a galactose-curable plasmid introduced expressing PFK1, CDC19, TKL1 and TAL1 under their native promoters, before performing two sequential rounds of CRISPR-mediated genome engineering to delete PCK1, TKL1 and TAL1, and knock-down CDC19 and PFK1 using the weak promoters RNR2 and REV1, respectively (Figure 2A). Moreover, several enzymes..

Minor comments:

9. References: Check format. Organisms in italics, page 28 shows some boxes lines 951-952

>> Organism names in the reference list are now in italics.

Furthermore, dashes present in the reference titles were changed into boxes by the reference manager. These changes have now been reverted.

10. The reference (Radivojević et al) provided for the ART algorithm is not a peer-reviewed article. Please update if possible.

>> The ART manuscript is under review in *Nature Communications* (NCOMMS-20-06356). The submitted version is available in the arxiv preprint. DOI from the peer-reviewed manuscript will be inserted when available.

11. Line 341: For(?) construction….

>> We are little confused about this, but have updated the text we think the reviewer refers to:

Discussion:
..ii) included the efficient one-pot construction of a library with different promoter combinations controlling the expression of these genes, and iii) used

12. Line 636: The success … was….

>> "were" changed into "was"

13. Line 595: Check ref Jessop

>> The reference title contained dashes which were changed into boxes by the reference manager. These changes have now been reverted.

14. Resolution of figures in supplementary should be improved

>> Ok. Font sizes in Figures S2-S6 have been increased by at least 2 points.

Reviewer #4 (Remarks to the Author):

This manuscript first used a genome-scale model (GSM) to predict 5 genes related to tryptophan synthesis in yeast. And then, with the help of CRISPR/Cas9 genome engineering tools, 6 promoters with different strengths were used to drive these genes to build a

combinatorial library with 7,776 (6^5) members. To facilitate high-throughput test of the library, a tryptophan biosensor was constructed with a dynamic range of 5 folds, and an operational range of ~2-200 mg/L trp. With the genotypic combinatorial library and phenotypic testing tool in hand, 507 colonies were tested separately, with output of >144,000 data points. These data were then used to predict the combination with optimized trp productivity; here commonly used ART model and EVOLVE algorithm were used. The new recommended designs improving tryptophan production by up to 17% compared to the best designs used for algorithm training. Combining GSMs with machine learning is certainly a promising approach and future direction of engineering cellular metabolism. This work is thus of certain interest. However, the significance of results and findings from this work is quite limited for the specific case of trp biosynthesis.

>> We are happy that the reviewer acknowledges the promise of the adopted approach for future direction of metabolic engineering.

We agree that the results presented are limited to the testbed of tryptophan biosynthesis. Yet, we consider this first example of combining mechanistic and mathematical modeling as of relevance to any other metabolic engineering effort, as long as the metabolic target and/or end-product can be monitored in high-throughput (e.g. RapidFire MS, biosensor, colorimetric, etc). Also, as we have now emphasized more clearly in the revised Discussion, our study has revealed several non-intuitive strain designs among the best-performing. These designs are directly transferable to metabolic engineering efforts for aromatics production in yeast, and potentially other microbes.
Having said this, and as can be seen from our response to Reviewer #3 (comments #6-7) further bioprocess optimization and metabolic engineering is relevant to pursue based on the best-performing machine learning-guided predictions from a relatively small metabolic engineering space (5 genes, transcriptional regulation only, feed-back inhibition). We thus think that the method presented in this study should be easily transferable/scalable to different hosts, pathways and molecules. We hope the reviewer following the read of our responses to Reviewer 3, as well as our responses to this reviewer's comments given below, would agree with us.

There are some shortcomings and unclear points in this manuscript:

1. Data size. As stated in the manuscript: "Following transformation, we randomly sampled 480 colonies from the library, together with 27 colonies from the five control strains (507 in total), and successfully cured 423", considering the repeated genotype (3.7% repeat showed Fig. 2B), only ~400 genotype-phnotype association data at different cultivation time were acquired. The claim that >144,000 data points, 1,728 isoclonal designs were used is misleading. The authors should state this point more clearly.

   >> We agree, and we are sorry for the lack of clarity. We have now added more explicit text related to the data size used for assessing the optimal sampling time-point, and the number of strains (and corrected number of data points) which were included as data for training the algorithms. The following two sections have been updated:

   Results (Engineering a tryptophan biosensor for high-throughput library characterization):
   ….with the intention to define optimal data sampling time point. To do so, we measured time-series data of OD and GFP at 82 time-points in triplicates for all 507 colonies (that is 480 from the library and 27 from the control strains), covering a total of 124,722 data points (Figures S4-S5).

and

Discussion:

To gather the large high-quality data set required for machine learning approaches, we developed a biosensor which enabled the sampling of >124,000 GFP intensity measurements (82 time points) as a proxy for tryptophan flux for 1,521 isoclonal designs (3 replicates x 507 strains) in a high-throughput fashion, of which data from 250 strains were eventually used for successful training of ML algorithms (Figures 3E, S5A).

Furthermore, we kindly ask the reviewer to see our answers to Reviewer 1, comment #1, as well as Reviewer 2, comment #1, also touching upon the need for clarification on data size and calculations.

2. Description. Page 9, lines 283-286. The sentence "As the quality ….." is not clear or ill-structured.

   >> We agree. We have made the following changes to the sentence:

   Results (Using machine learning to predict metabolic pathway designs):

   As the quality of the data is of paramount importance for machine learning predictions, data was initially filtered in order to avoid strains i) with insufficient growth, ii) without sequencing data, iii) with incorrect assembly, iv) without plasmid curation, or v) which exhibited more than one genotype (see METHOD; Figure S5).

3. Prediction power. Although "an order of magnitude higher number of strains than in previous machine learning-guided metabolic engineering studies (Alonso-Gutierrez et al., 2015; Lee et al., 2013a; Redding-Johanson et al., 2011; Zhou et al., 359 2018a)", the prediction power seems to be not very good. Why? The quality of the training dataset?

   >> First, we would like to kindly ask the reviewer to see our response to the largely overlapping comment #12 posed by Reviewer 1. Secondly, different problems present different levels of difficulty when being "learnt". Easy problems can be learnt with small amounts of data, while difficult problems need much larger data sets. The difficulty of a problem depends on a myriad characteristics (e.g. coupling of host and pathway, presence of tight regulation, pathway length, etc.), but the only way to ascertain the difficulty of a problem is by checking how the predictive power of the algorithms improves with more data (i.e. more DBTL cycles). This aspect is also discussed in the ART manuscript (Radivojević et al, see also preprint in https://arxiv.org/abs/1911.11091)

   Could iterative prediction strategy by feeding the predicted data to the initial library work?

   >> Indeed. More DBTL cycles in which the data corresponding to the recommendations is added to the training library would definitely help, as it is shown to be the general case in both Radivojević et al, https://arxiv.org/abs/1911.11091) and Borkowski et al. https://www.biorxiv.org/content/10.1101/751669v1). However, in this study we sought to "stress-test" the predictive power generated from a large high-quality data set, and use recommendations based off of this first cycle to assess the predictive power. This we already stated in the Discussion of the first manuscript version:

   Discussion:

However, if subsequent design-build-test-learn cycles are performed, the diversity of recommendations of the explorative approach should help avoid local optima of tryptophan production (Figure 4E-F).

And we have further followed up upon this in our revised manuscript

Discussion:
This could be used to argue that more engineering iterations on even smaller data sets, potentially coupled to mixed exploitation and exploration approaches as recently demonstrated for cell-free production[68], should be a valid avenue for ML-guided engineering of even less genetically-tractable chassis, and for which no high-throughput screening method may even exist.

Could published models used in previous study yield better results? The authors should address these points or give comments on them.

>> Maybe - see also our answer to the first part of the comment #3. In our study we trained a multitude of algorithms to come to final assemble model predictions. These models we consider performing reasonably well on data never seen before (Figure 4E-F). Furthermore, we have tested the ART and EVOLVE model approaches in explorative and exploitative modes, respectively, in order to assess the "landscape" of recommendations put forward by the two modelling approaches when run in different modes. Based on this we have updated the Discussion of our revised manuscript:

Discussion:
With regards to this, we performed a follow-up test running the ART and EVOLVE approaches in explorative and exploitative modes, respectively. Here, we observed that the recommendations from EVOLVE in exploitative mode had overlaps of 20% (6/30) and 23% (7/30) to ART recommendations in exploitative and EVOLVE recommendations in explorative mode, respectively. Complementary to this, the recommendations from ART in explorative mode only had overlaps of 3% (1/30) and 0% (0/30) to ART recommendations in exploitative and EVOLVE recommendations in explorative mode, respectively (Figure S8), indicating that the uncertainty of prediction of high GFP synthesis rate weighted differently for the two models in explorative mode.

We hope that our careful referencing to the few relevant studies on this subject, as well as newly performed modelling approaches in this revised manuscript illustrates the credibility of our work and the derived take-homes.

4. Knowledge learned from the data? Which combinations give better productivity? Are there any rules or explanation behind it?

   >> In this paper, we have shown how to use prior biological knowledge (genome-scale model) to guide and improve ML predictions. In terms of combinations improving our knowledge about metabolic regulation of aromatics biosynthesis in yeast, our findings have enabled us to both corroborate earlier discoveries (e.g. low expression of *CDC19*), but also to highlight completely new non-intuitive combinations (e.g. low expression of *TKL1* and high expression of *TAL1*. This we have now also reflected more explicitly on in the Discussion of the revised manuscript.

Discussion:

Surprisingly, the top-5 strains predicted to have high tryptophan biosynthesis rates (SP606, SP616, SP624, SP588 & SPSP620, Tabel S8) all had low expression of TKL1 and high expression of TAL1,..

Further, to distill biological knowledge from ML results is an area of active research (e.g. explainable AI, XAI), but also regarded as beyond the scope of this paper. Also, while the algorithms presented in this study provide predictive power for tryptophan biosynthesis rates which can be used to distill rules for other projects, there is no systematic manner to do this yet.

5. Could these data help to optimize the genome scale model?

>> GSMs represent the collection of all stoichiometrically feasible states that the cell can achieve; therefore, the results from this study would not be of use for improving predictions from traditional GSMs, as each experimental phenotype is already included within the simulated solution space. However, the results would definitely be helpful for models that account for additional levels of information, such as metabolic/expression hybrid models (https://doi.org/10.1016/j.copbio.2014.12.017) or the recently published whole-cell model of yeast (https://doi.org/10.1002/bit.27298). For example, with the experimental results from this study, the feasible kinetics in the aforementioned models could be further constrained. However, this is deemed beyond the scope of this study.

6. The conclusion cannot be fully supported by the data, e.g. “ultimately producing a total increase of 106% in tryptophan accumulation compared to optimized reference designs”, they even did not give the data of “real” tryptophan concentration, only GFP fluorescence intensity. This problem seems to be severe when considering the strong scattering of the seemingly linear relationship between fluorescence intensity and extracellular tryptophan concentration showed in Fig. 3D.

>> We agree that this should be rephrased to ensure that it is clear that the increase is related to GFP biosynthesis rate. This issue was also touched upon by Reviewer 3 in comment #2 (see above). To clarify, the ART model recommended strain ID SP606 to have high GFP biosynthesis rate. Indeed, compared to reference strain ID SP507 (wild-type promoters and feedback resistant), strain SP606 had a synthesis rate of 298.3 MFI/hr compared to the synthesis rate of SP507 being 144.8 MFI/hr (106%, see also new Table S12). To put this into context of tryptophan, we have now measured extracellular tryptophan concentration by HPLC from 24 hrs small-scale batch cultivations (see new Figure S7). Here we found that the best performing ML-guided recommended strain design accumulated 74% higher tryptophan titers at this time-point compared to the best design used for algorithm training (SP271). We have now updated the Abstract, Introduction, Result and Discussion accordingly - see all updated text in the response to Reviewer 3, commnet #2 above. .

Furthermore, the Trp concentration (a few mg/L) achieved is very low and the improvement is marginal.

>> While we agree that the tryptophan concentrations reported from the strains cultivated in small-scale batch cultivations (i.e. non-optimized bioprocess conditions) are much lower than state-of-the-art titers derived from heavily engineered yeast cell factories for aromatics-derived production (>10 g/L)(Averesch and Krömer 2018; Liu et al. 2019), we consider the demonstration to improve tryptophan biosynthesis by the use of mechanistic and machine learning models, based on a single DBTL cycle and a limited number of gene targets, far from “marginal” (74% increase in titer and 43% increase in productivity from a single DBTL cycle). We hope the reviewer acknowledges that this study is a first demonstration of this

concept, and as stated in our response to this reviewer's comment #1, we believe this modeling concept should be ready to scale to more engineering targets and even other platform strain optimization regimes.

7. Novelty of the findings. In fact, the genotypes (knock-downs of both CDC19 and PFK1, low expression of TKL1 and high expression of TAL1) of the best performing strains (SP606, SP616) predicted by machine-learning are more or less known in the literature or can be relatively easily inferred from the pathways by considering the fact that PEP and E4P are two important precursors of trp synthesis.

   >> Here we disagree. While it is true that we focused on (model-guided) gene targets perturbing E4P and PEP pools, we think it is critical to emphasize that even if the machine learning algorithms trained in this study predicted more or less rational designs known in the literature, in our study this was accomplished only by "looking" at the data obtained, and not having any knowledge about the system whatsoever. This also means that our approach is promising for optimizing systems for which such prior knowledge is not available. Furthermore, we would like to state that we have found no literature disclosing the testing of combinations giving the best strain designs from this study; for instance our "top-5 strains predicted to have high tryptophan biosynthesis rates (SP606, SP616, SP624, SP588 & SPSP620, Tabel S8) all had low expression of *TKL1* and high expression of *TAL1*, despite the report that overexpression of *TKL1*, rather than *TAL1*, leads to higher aromatic amino acid production in both *E. coli* and yeast[36,76]", as we now also state in the revised Discussion (please, also see our response to comment #4). While we do not question the "metabolic rules-of-thumb" based on single gene edits, the power of the approach adopted in this study lies in the modelling of combinatorial gene edits not performed before. Having said this, we obviously believe that a logical next step to further improve shikimate flux in the future would be to combine the *bonafide* new data-driven learnings from this large data set with prior art.

8. Target genes. It should be mentioned that in addition to PEP and E4P several other metabolites like glutamine, serine and 5-P-D-ribose-diphosphate are direct or indict precursors of trp synthesis. Genes related to the formation of these metabolites should be also considered.

   >> We agree that there are many more targets than the ones we have investigated here that would be interesting to test. Indeed, using FBA we sought to rank gene targets for this study, including exactly genes related to some of the metabolites/pathways listed by the reviewer (Table S5). We see that following the synthesis of aromatic amino acids, FBA also predicts glycine, serine and threonine metabolism to impact flux towards tryptophan, while pathways towards pyrimidine (which includes 5-P-D-ribose-diphosphate) and alanine/aspartate/glutamate (which includes glutamine) metabolism are ranked lower (see Table S5). Having said this, we (as in any other study of this complex metabolism), have needed to prioritize the gene targets going into our experimental design for the study, as also reflected by our wish to rank the FBA-derived results into KEGG pathways. We hope the reviewer acknowledges that the combinatorial designs obtained in this study can be considered a platform from which both more model-guided iterations can extend from and into which further gene targets can be included. This is indeed the kind of thinking we hope to inspire by our study, yet beyond the scope of the extensive design space (already 7,776 design possibilities) already mined in this study.

   The real challenge for efficient trp synthesis is dynamically balancing the synthesis of these precursors depending on growth rate, the desired trp yield and productivity. The export of trp is also very crucial. The GSMs and machine learning algorithms cover only a small part of a very

complex metabolism and the regulations (many!) are not considered at all. Some more recent advanced studies on the metabolic engineering of the trp pathway should be considered. Very high titer, yield and productivity of trp have been achieved by rational metabolic engineering.

Firstly, we do agree that GSMs have limitations in their predictions, as they do not account for processes such as regulation and molecular crowding during membrane transport. To make these limitations more visible we have mentioned in the revised Discussion alternative modeling approaches that account for more biological layers and could be employed in follow-up studies

Discussion:
Without any experimental input, GSMs are able to guide metabolic engineering using various constraint-based algorithms, which, however, predict a large number of potential targets and may also miss some effective ones, e.g. PFK1 in our study. This could be due to the lack of other information beyond metabolism, e.g. regulation in GSMs. To address this problem, manual efforts are currently needed to filter out less relevant targets, and add intuitively promising ones based on existing knowledge and literature mining. Additionally, applying our approach to new models that enhance GSMs with more levels of information, such as kinetics[78], gene expression[79] and regulation[80] is envisioned to further improve gene target selection in future studies.

Secondly, and as already mentioned in lines 111-112 and 408 of the original manuscript, tryptophan metabolism is indeed heavily regulated. While the goal in this study was to show the efficacy of the combined modeling approach, and not *per se* aiming to get to record tryptophan productivities, several additional and more recent studies founded on mechanistic and rational strategies for aromatics production optimization are indeed relevant to highlight and discuss. In our revised Discussion we have now added more citations to recent metabolic engineering studies reaching high titers, rates and yields of products derived from engineering the complex regulated aromatics metabolism, and also more explicitly now elaborate on our main findings in relation to such prior art:

Discussion:
Indeed, in bacteria, pyruvate kinase knockout has been used for the overproduction of shikimate-pathway derived aromatics products in bacteria[71–73]. Likewise, since yeast cells with CDC19 deletion cannot grow on glucose[74], dynamic silencing of CDC19 and PYK2 have been used for boosting production of para-hydroxybenzoic acid (PHBA)[75], just as expression of a mutant CDC19 pyruvate kinase with seemingly lower activity, in combination with overexpression of transketolase (TKL1), have been demonstrated to improve 2-phenylethanol (2PE) production in yeast[76]. On the contrary, a similar strategy with lower CDC19 activity, but in combination with zwf1Δ deletion (lacking the committed step towards the oxidative branch of PPP) was shown to reduce tyrosine titers[77]. Surprisingly, the top-5 strains predicted to have high tryptophan biosynthesis rates (SP606, SP616, SP624, SP588 & SPSP620, Tabel S8) all had low expression….

9. Biosensor. The existence of biosensor in the cell will have influences on tryptophan production, it would be better to test the tryptophan production without the biosensor plasmid.

>> This is a fair point to raise, especially had the end goal of this study been to benchmark obtained titers and productivities with prior art - which is not the case. Moreover, in a previous study of ours we used exactly the same weak *REV1* promoter, used in this study for

driving the expression of the TrpR-based tryptophan biosensor, to drive expression of the *cis,cis*-muconic acid biosensor BenM. In that study we found only marginal, yet not significant, effect when comparing *cis,cis*-muconic acid titers of engineered yeast cells with or without the biosensor expressed (Snoek et al. 2018). Lastly, while the biosensor was a key part of the study in order to produce the large amounts of data needed to feed the ML approaches, the biosensor would never be an integral part of a cell factory for high aromatics production, unless as part of a regulatory control circuit (Williams et al. 2015; Rugbjerg et al. 2018). For all of the above, we did not delete the biosensor before tryptophan quantifications. We hope the reviewer finds these previous observations and considerations satisfactory to justify our choice of analysis.

REVIEWERS' COMMENTS:

Reviewer #1 (Remarks to the Author):

I am generally happy with the answers to my questions/comments and the modifications made in the revised manuscript. In particular, I am pleased to see that now both ART and EVOLVE are used in exploitation and exploration modes. I just have one remaining question: how the 6 strains listed in Figure S7 were chosen? Are these parts of the 8 recommendations found in the top-ten productivity by ART and EVOLVE?

Reviewer #2 (Remarks to the Author):

All my comments and questions have been properly addressed – only the promised reduction of the platform strain/library part was not really a big leap forward. However, if the other reviewers feel that their questions have been answered, this manuscript should be accepted for publication.

Reviewer #3 (Remarks to the Author):

I thank the authors for clarification of my points and congratulate them on a nice piece of work. I recommend publication.

[Editor: We asked Reviewer #3 to comment your response to Reviewer #4's suggestions. Reviewer #3 states in Remark to Editor section that (s)he thinks Reviewer #4 previous suggestions have been addressed.]
